# Obesity and diet independently affect maternal immunity, maternal gut microbiota and pregnancy outcome in mice

**DOI:** 10.3389/fimmu.2024.1376583

**Published:** 2024-07-12

**Authors:** Lieske Wekema, Sam Schoenmakers, Nicole Schenkelaars, Anne Laskewitz, Lei Liu, Lisa Walters, Hermie J. M. Harmsen, Régine P. M. Steegers-Theunissen, Marijke M. Faas

**Affiliations:** ^1^ Department of Pathology and Medical Biology, University Medical Center Groningen, University of Groningen, Groningen, Netherlands; ^2^ Department of Obstetrics and Gynaecology, Erasmus University Medical Center, Rotterdam, Netherlands; ^3^ Department of Medical Microbiology, University Medical Center Groningen, University of Groningen, Groningen, Netherlands; ^4^ Department of Obstetrics and Gynaecology, University Medical Center Groningen, University of Groningen, Groningen, Netherlands

**Keywords:** pregnancy, maternal obesity, gut microbiota, immune response, diet

## Abstract

**Introduction:**

Maternal obesity poses risks for both mother and offspring during pregnancy, with underlying mechanisms remaining largely unexplored. Obesity is associated with microbial gut dysbiosis and low-grade inflammation, and also the diet has a major impact on these parameters. This study aimed to investigate how maternal obesity and diet contribute to changes in immune responses, exploring potential associations with gut microbiota dysbiosis and adverse pregnancy outcomes in mice.

**Methods:**

Before mating, C57BL/6 mice were assigned to either a high-fat-diet (HFD) or low-fat-diet (LFD) to obtain obese (n=17) and lean (n=10) mice. To distinguish between the effects of obesity and diet, 7 obese mice were switched from the HFD to the LFD from day 7 until day 18 of pregnancy (“switch group”), which was the endpoint of the study. T helper (Th) cell subsets were studied in the spleen, mesenteric lymph nodes (MLN) and Peyer’s patches (PP), while monocyte subsets and activation status were determined in maternal blood (flow cytometry). Feces were collected before and during pregnancy (day 7,14,18) for microbiota analysis (16S rRNA sequencing). Pregnancy outcome included determination of fetal and placental weight.

**Results:**

Obesity increased splenic Th1 and regulatory T cells, MLN Th1 and PP Th17 cells and enhanced IFN-γ and IL-17A production by splenic Th cells upon ex vivo stimulation. Switching diet decreased splenic and PP Th2 cells and classical monocytes, increased intermediate monocytes and activation of intermediate/nonclassical monocytes. Obesity and diet independently induced changes in the gut microbiota. Various bacterial genera were increased or decreased by obesity or the diet switch. These changes correlated with the immunological changes. Fetal weight was lower in the obese than the lean group, while placental weight was lower in the switch than the obese group.

**Discussion:**

This study demonstrates that obesity and diet independently impact peripheral and intestinal immune responses at the end of pregnancy. Simultaneously, both factors affect specific bacterial gut genera and lead to reduced fetal or placental weight. Our data suggest that switching diet during pregnancy to improve maternal health is not advisable and it supports pre/probiotic treatment of maternal obesity-induced gut dysbiosis to improve maternal immune responses and pregnancy outcome.

## 1 Introduction

The general prevalence of obesity has significantly increased in the past decades (1), including in women during the reproductive age (2). Obesity during pregnancy is an important health issue as it is associated with increased risks of adverse pregnancy outcomes for both mother and child, such as gestational diabetes mellitus, preeclampsia, an increased risk for congenital malformations, macrosomia or fetal growth restriction, as well as obesity later in life (3–6). However, the underlying mechanisms by which obesity affects maternal and fetal outcomes are still largely unknown.

During healthy pregnancy, unique adaptations occur in the maternal immune system in order to tolerate the semi-allogeneic fetus, while immunological protection against pathogens is maintained (7). Various changes are observed in the peripheral maternal immune response, including a decreased T helper (Th) 1 versus Th2 ratio (8), a decrease in Th17 cells (9), an increase in regulatory T (Treg) cells (10), increased activation of monocytes (as measured by CD64 and CD80 expression), an increase in non-classical monocytes and a decrease in classical monocytes (11–13). Aberrant adaptations in the maternal immune response are associated with adverse pregnancy complications like miscarriage, preeclampsia and prematurity (14, 15). Numerous animal and human studies have shown that obesity is associated with low-grade inflammation and other immunological changes (16–20). However, to date, it is relatively unknown how maternal obesity impacts the maternal immune response. Studies investigating this topic indicate that pregnant women with pre-pregnancy obesity have increased serum levels of pro-inflammatory cytokines, such as IFN-ɣ and TNF-α (21–23). Another study described a disruption of the Th1/Th2/Th17 axis in the periphery of women with obesity. This was caused by an enhanced Th1/Th2 ratio and increased Th17 response with higher maternal BMI (24). These data suggest a causal link between maternal obesity, immune anomalies, and adverse pregnancy outcomes.

Factors involved in pregnancy-related immune adaptations include enhanced sex hormone production such as progesteron, and placental signals, such as cytokines and extracellular vesicles (25). Recently, we and others have shown that immune adaptations during pregnancy are also influenced by the maternal gut microbiota during pregnancy (26, 27). Gut microbiota have been shown to produce a variety of immunomodulatory molecules, such as short-chain fatty acids (SCFAs), that can influence intestinal and peripheral immune responses (28). Also, direct interaction of microbiota with gut epithelial or intestinal immune cells have been shown to influence intestinal immunity and peripheral immunity (29). The observed changes in the maternal gut microbiota during healthy pregnancy (26, 27) may therefore be essential to induce and maintain necessary maternal immune adaptations. Since some studies have shown that the maternal gut microbiota differs between pregnant women with or without obesity (30–32), obesity-induced gut microbiota dysbiosis may induce anomalies in immune adaptations and contribute to adverse pregnancy outcomes. More insight in when and how this happens might lead to new ways for intervention and prevent obesity-induced changes in immunity and microbiota composition.

The gut microbiota is shaped in early life and becomes more definitely established in adulthood. Its composition depends on age, ethnicity, medication use, and most importantly on diet (33). The diet directly influences the composition and activity of the gut microbiota, and dietary adjustments can already induce specific microbial shifts within the gut microbiota within 24 hours (34). Individuals with obesity most often consume a high-calorie “Western” diet that is enriched in saturated- and trans fats as compared to lean individuals (35). Diet may indirectly affect the immune system through microbial changes (36), but also affects circulating nutrients that are directly involved in the immune response, such as fatty acids (37). Therefore, it is challenging to determine whether the state of obesity itself or the dietary pattern or a combination thereof is responsible for observed immunological changes in obesity.

As it is relatively unknown which immune adaptations occur in maternal obesity and whether maternal immune adaptations in obesity are due to dietary patterns or dysbiosis in gut microbiota, we designed the current mouse study. We hypothesize that maternal obesity induces derangements in immunity, primarily mediated by obesity-induced gut dysbiosis, which in turn induces adverse pregnancy outcomes. For this purpose, we used obese (induced by high-fat diet (HFD)) and lean pregnant mice (fed a matched low-fat diet (LFD)). To evaluate the effect of the diet, one group of obese pregnant mice was switched to the LFD on day 7 of pregnancy. In all groups we determined maternal peripheral and intestinal immune responses (day 18), maternal gut microbiota composition (pre-pregnancy, day 7, 14 and 18) and pregnancy outcome (day 18), as well as the correlation between the immune response and the gut microbiota (day 18) and the correlation between the immune response and pregnancy outcome (day 18).

## 2 Materials and methods

### 2.1 Study design

**Figure 1:**
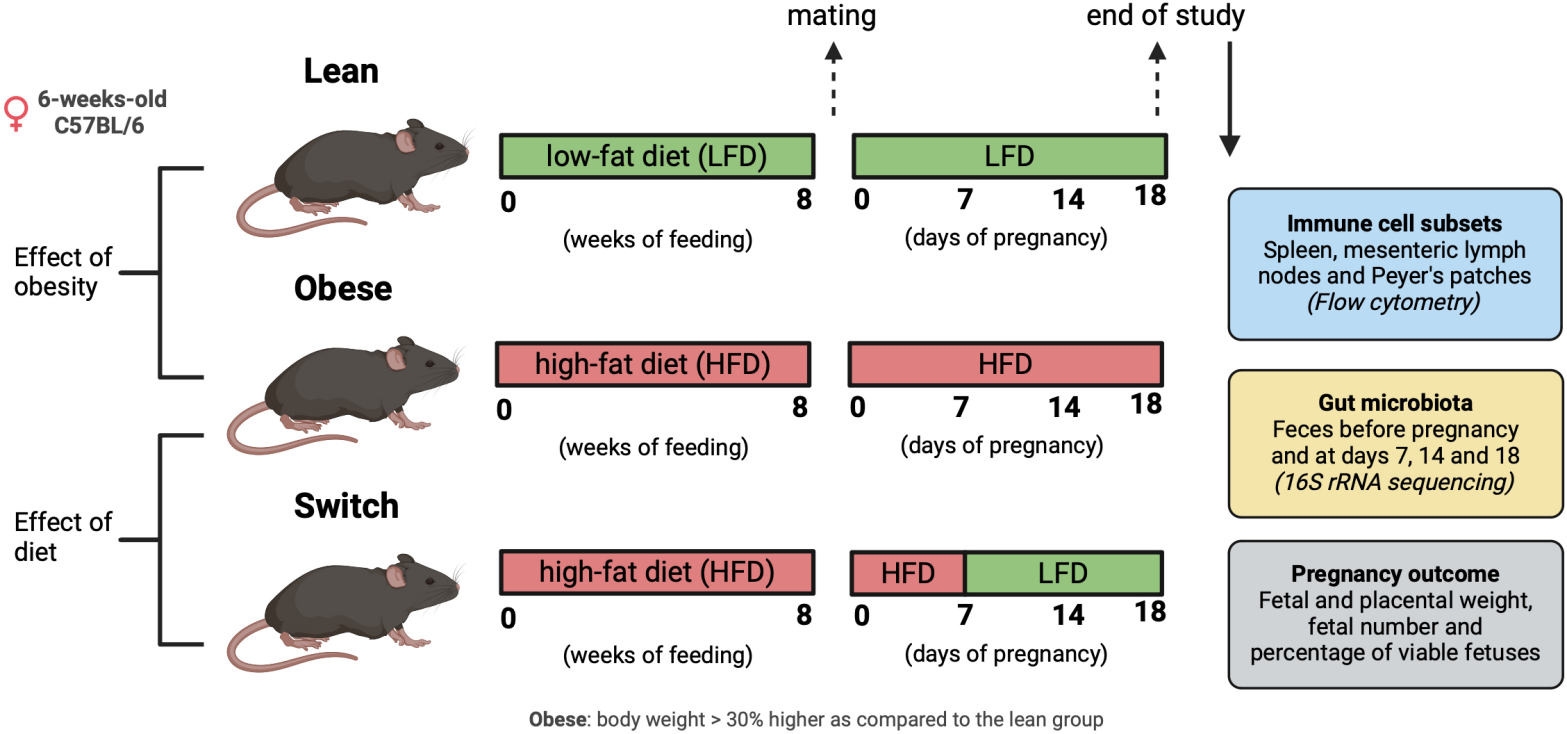
Graphical schematic of experimental approach. Created with BioRender.com.

Obesity was induced by means of a standardized HFD; a standard approach often used in literature to induce obesity in rodents (38). Lean control mice received a matched LFD. Next, obese and lean mice were rendered pregnant. During pregnancy, mice in the obese and lean groups were maintained on the HFD and LFD, respectively. To determine whether obesity or the HFD is responsible for immune alterations in pregnancy, we also included a so-called ‘switch’ group: after obesity-induction by the HFD, pregnant mice were switched to the LFD at day 7 (E7) of pregnancy. This was done to discriminate between the effect of obesity perse and the effect of eating a HFD during pregnancy (**Figure 1**). In the 3 groups of mice, we investigated the changes in the gut microbiota composition before pregnancy and at E7, E14 and E18 of pregnancy (similar to the end of the first, second and third trimester in humans), and maternal immune responses only at E18. We also determined the correlation between the immune response and the gut microbiota during pregnancy at E18. Finally, pregnancy outcome included measurement of fetal and placental weight, total number of fetuses and the percentage of viable fetuses at E18. We also determined the correlation between fetal/placental weight and the immune response at E18.

### 2.2 Animals

All animal experiments performed in this study were approved by the Central Committee on Animal Experimentation in the Netherlands (CCD application number AVD10500202010704). Female and male C57BL/6JOlaHsd mice (Envigo, Horst, The Netherlands) were obtained at an age of 6-weeks-old. Upon arrival in the facility, females were randomized based on body weight and assigned to either a HFD (60 kcal% fat, D124921i, Research Diets Inc., New Brunswick, USA) or an ingredient-matched LFD (10 kcal% fat, D12450Ji, Research Diets Inc.) for 8 weeks to respectively obtain obese and lean mice. Female mice were considered obese when their body weight was 30% higher as compared to the lean group (**Supplementary Figure 1**). All female mice were co-housed in isolated ventilated cages (5 mice per cage) with a 12/12h light/dark cycle and ad libitum access to water and food. Male mice were housed individually under equal circumstances and received the LFD. After 8 weeks on the diet, vaginal smears were taken to check the ovarian cycle, and when in proestrus, females were placed with a male overnight. During mating, male mice were offered the same diet as their female counterpart. The next morning, females were checked for a vaginal plug, and this day was considered E0 of pregnancy. During pregnancy, mice in the obese and lean groups were maintained on the HFD and LFD, respectively. Mice in the switch group changed from the HFD diet to the LFD at E7 of pregnancy. Feces was collected from all 3 groups, before and during pregnancy on E7, E14 and E18 by placing the mice individually in an empty sterile cage for a short period of time until defecation. Immediately after defecation, fecal samples were frozen in liquid nitrogen and stored at -80°C until microbiota analyzes. Maternal body weight was monitored during pregnancy (**Supplementary Figure 2**). Females were sacrificed under general anesthesia (isoflurane/O2) at E18 of pregnancy by means of exsanguination via a cardiac puncture. Blood was collected in EDTA tubes (BD Biosciences, Breda, The Netherlands) and spleens, mesenteric lymph nodes (MLN), and Peyer’s patches (PP), were harvested and stored on ice in RPMI medium (Thermo Fisher Scientific, Waltham, USA) + 10% decomplemented fetal calf serum (dFCS; Thermo Fisher Scientific) until cell isolation within 2 hrs. Also, fetal weight, placental weight, fetal number and the number of viable fetuses were determined.

### 2.3 Cell isolation from spleen, mesenteric lymph nodes and Peyer’s patches

Spleens, MLNs and PPs, were cleaned with tweezers to remove pieces of other tissues. MLNs and PPs were mechanically disrupted between two microscopy glasses in 2 ml RPMI medium + 10% (v/v) dFCS, whereafter the suspensions were filtered by means of polystyrene tubes with cell strainer caps (35 µm pores; Corning, Amsterdam, The Netherlands) to obtain single cell suspensions. Spleens were first cut into small pieces, whereafter the pieces were mechanically disrupted between two microscopy glasses in 3 ml RPMI medium + 10% (v/v) dFCS. Subsequently erythrocytes were eliminated by incubation with 4 ml ice-cold ammonium chloride buffer (0.16M NH_4_Cl, 0.01M KHCO_3_, 0.03mM CH_3_COONa-2H_2_O). After centrifugation at 600x *g* for 3 min at 4 °C, splenocytes were washed with FACS buffer (2% (v/v) dFCS in 1x DPBS (Thermo Fisher Scientific)). After another washing step, splenocytes were resuspended in 2 ml RPMI medium + 10% (v/v) dFCS, whereafter the suspensions were filtered by means of the polystyrene tubes with cell strainer caps. For spleens, MLNs and PPs, cell concentrations were determined with a Z1 coulter particle counter (Beckman Coulter Life Sciences, Brea, USA). All steps were performed on ice. Isolated cells were used for T cell staining. In addition, leftover splenocytes were frozen in 10% DMSO (in dFCS) and stored at -80°C, until cytokine production was assessed.

### 2.4 T cell staining

Cells from the spleen, MLNs and PPs were stained for T helper cell populations. Per sample approximately 1 x 10^6^ cells were added to a 96 wells plate (Corning), and after centrifugation at 600x *g* for 3 min at 4°C, cells were washed in 200 µl DPBS. Next, cells were resuspended and incubated in 100 µl Zombie NIR viability solution (1:1000, cat #423105, Biolegend, San Diego, USA) for 30 min at room temperature (RT). After centrifugation, cells were washed twice in 200 µl FACS buffer. Next, cells were resuspended and incubated in 50 µl extracellular blocking medium (78% (v/v) FACS buffer, 20% (v/v) rat serum (Jackson, Newmarket, UK) and 2% (v/v) purified anti-mouse CD16/32 (cat #101302, Biolegend)) for 10 min to prevent non-specific binding of antibodies. After centrifugation, cells were resuspended and incubated in 25 µl extracellular antibody mix for 30 min on ice (**Supplementary Table 1**). After incubation and two rounds of washing in respectively 150 and 200 µl FACS buffer, cells were fixed in 200 µl FACS lysing solution (1x, BD Biosciences) for 30 min on ice. After two washing steps with 200 µl permeabilization buffer (1x, eBioscience, Vienna, Austria), cells were resuspended and incubated in 50 µl intracellular blocking medium (80% (v/v) permeabilization buffer and 20% (v/v) rat serum) on ice for 10 min. After centrifugation, cells were resuspended and incubated in 50 µl intracellular antibody mix for 30 min on ice (**Supplementary Table 1**). After two rounds of washing with respectively 150 and 200 µl permeabilization buffer, cells were resuspended in 200 µl FACS buffer and stored at 4°C until data acquisition within 24 hours. Washing steps included centrifugation at 600x *g* for 3 min at 4°C and all steps were performed in the dark after the addition of the viability solution.

### 2.5 Cytokine production by splenic T helper cells

Splenic T helper cells were stimulated *ex vivo* to assess cytokine production. Frozen splenocytes were thawed in a water bath (37 °C) until a small ice clump remained, whereafter the splenocyte suspensions were added to 13 ml ice-cold RPMI + 10% dFCS. After centrifugation at 600x *g* for 3 min at 4 °C, cells were resuspended in 2 ml pre-heated (37 °C) RPMI + 10% dFCS, after which cell concentrations were determined with a Z1 coulter particle counter. Per sample approximately 1.5 x 10^6^ cells were added to a 96 wells plate, and after centrifugation at 600x *g* for 3 min at RT, splenocytes were stimulated with 50 µl stimulation mix (Phorbol 12-myristate 13-acetate (Sigma, 20 ng/ml), ionomycin (Sigma, 1.8 µg/ml) and Brefeldin A (Sigma, 10 µg/ml) in RPMI + 10% dFCS) for 4 hours at 37 °C. After two rounds of washing in respectively 150 and 200 µl DPBS, cells were resuspended and incubated in 100 µl Zombie NIR solution (1:1000) for 30 min at RT. After centrifugation, cells were washed twice in 200 µl FACS buffer. Next, cells were resuspended and incubated in 50 µl extracellular blocking medium to prevent non-specific binding of antibodies. After centrifugation, cells were resuspended and incubated in 25 µl extracellular antibody mix for 30 min on ice (**Supplementary Table 2**). After two rounds of washing in respectively 150 and 200 µl FACS buffer, cells were fixed in 200 µl FACS lysing solution (1x) for 30 min on ice. After two washing steps with 200 µl permeabilization buffer (1x), cells were resuspended and incubated in 50 µl intracellular blocking medium on ice. After centrifugation, cells were resuspended and incubated in 50 µl intracellular antibody mix for 30 min on ice (**Supplementary Table 2**). After two rounds of washing in respectively 150 and 200 µl permeabilization buffer, cells were resuspended in 200 µl FACS buffer and stored at 4°C until data acquisition within 24 hours. Washing steps included centrifugation at 600x *g* for 3 min at 4°C and all steps were performed in the dark after the addition of the viability solution.

### 2.6 Monocyte staining

Maternal EDTA blood was stained for monocyte subsets and activation status. First, 200 µl blood was mixed with 200 µl RPMI medium + 10% (v/v) dFCS. Next, 50 µl of extracellular blocking medium (78% FACS-EDTA buffer (5% (v/v) dFCS and 372 µg/ml EDTA (Sigma) in DPBS), 20% (v/v) rat serum and 2% (v/v) purified anti-mouse CD16/32) was added for 10 min at RT. After a centrifugation step at 600x *g* for 3 min, cells were resuspended and incubated in 25 µl antibody mix (**Supplementary Table 3**) for 30 min at RT. After two washing steps with 500 µl FACS-EDTA buffer, cells were fixed, and erythrocytes were lysed in 1 ml FACS lysing solution (1x) for 20 min at RT. After three rounds of washing in 500 µl FACS-EDTA buffer, cells were resuspended in 200 FACS-EDTA buffer and stored at 4°C until data acquisition within 24 hours. Washing steps included centrifugation at 600x *g* for 3 min and all steps were performed in the dark after the addition of the extracellular antibody mix.

### 2.7 Flow cytometry

Data acquisition was performed on a NovoCyte Quanteon Flow Cytometer (Agilent, Santa Clara, USA) using NovoExpress software (Agilent). FCS Express software version 6 (*De Novo* Software, Pasadena, USA) was used for data analysis. Gating strategy was performed as described in **Supplementary Figure 3** (T helper cells), **Supplementary Figure 4** (cytokine production by splenic T helper cells) and **Supplementary Figure 5** (monocytes).

### 2.8 DNA isolation of fecal samples

DNA from murine feces was isolated according to the method described by Goffau et al. (39) and Yu et al. (40). For DNA isolation 10-300 mg of feces were added to sterile 2.0 ml screw cap tubes (BIOplastics, Landgraaf, The Netherlands) filled with 0.5 gram of zirconium silica beads (0.5 mm diameter; Biospec, Bartlesville, USA) and 4 glass beads (3 mm diameter; Biospec). Per tube, 1 ml of lysis buffer was added (500 mM NaCl, 50 mM Tris-HCl [pH 8.0], 50 mM EDTA and 4% (v/v) SDS), after which the samples were treated in a Precellys 24 tissue homogenizer (Bertin instruments, Montingny-le-Bretonneux, France) at 5.5 ms for 3 x 1 min with 30 second pauses in between at RT. Subsequently, samples were heated at 95 °C for 15 min, while shaking by hand every 5 min. After centrifugation at 16.000x *g* for 5 min at 4 °C, supernatant was collected in sterile 2.0 ml tubes (Eppendorf, Hamburg, Germany). To ensure the highest possible yield of DNA, 300 µl of fresh lysis buffer was added to the screw cap tubes, whereafter the described procedure was repeated. Next, 260 µl of 10 M ammonium acetate was added to each lysate to precipitate proteins during a 5 min incubation step on ice. After centrifugation at 16.000x *g* for 10 min at 4 °C, the supernatants were transferred into new sterile 2.0 ml tubes (Eppendorf). To ensure the removal of all proteins, lysates were treated a second time using the same procedure, whereafter the lysates were divided over two sterile 1.5 ml tubes (Eppendorf). Subsequently, nucleic acids were precipitated by the addition of one volume of isopropanol during a 30 min incubation step on ice. After centrifugation at 16.000x *g* for 15 min at RT, the supernatants were removed by decanting, whereafter the tubes were placed upside down to get rid of most of the moisture. Then, nucleic acid pellets were washed with 500 µl 70% ethanol for 2 min. After decanting, the tubes were airdried, and dissolved in 100 µl buffer AE (Qiagen, Hilden, Germany), whereafter they were stored overnight at 4 °C. The next day, aliquots were pooled, and RNA was removed by the addition of 1 µl DNase-free RNase (500 µg/ml, cat #11579681001, Roche, Basel Switzerland) for 15 min at 37 °C. Next, 15 µl proteinase K (Qiagen) and 200 µl buffer AL (Qiagen) were added, and after an incubation step of 10 min at 70 °C, the samples were placed on ice for 1 min. Then, 200 µl of ice-cold 100% ethanol was added, whereafter DNA purification was performed according to the protocol of the QIAmp DNA Mini Kit (Qiagen). DNA concentrations were measured with a NanoDrop ND-1000 spectrophotometer (Thermo Fisher Scientific). A negative DNA extraction control was taken along to monitor contamination of the reagents.

### 2.9 PCR, 16S rRNA gene sequencing, quality control and taxonomy assignment

The V3-V4 region of the 16S rRNA gene was amplified with modified barcoded 341F and 806R primers. Details on PCR, barcoded primers and sequencing library preparation were documented previously (41). The amplicons were sequenced by a Miseq Illumina sequencing platform. The paired-end reads, demultiplexed based on barcode, were retrieved from the Illumina platform and were instructed by EasyAmplicon analysis pipeline (42). The joined-reads were quality controlled with maximum error rate 1% and primer sequences were cut by VSEARCH (43). Denoising (removing chimeric sequences, removing singletons, and dereplication) was done with USEARCH and VSEARCH (44). The Amplicon Sequence Variants (ASVs) were assigned based on Ribosomal Database Project set 18 (45). A negative PCR and sequencing control were taken along to monitor contamination.

### 2.10 Statistical analysis

Statistical analysis on maternal body weight, weight gain during pregnancy, fetal weight, placental weight, fetal number, the percentage of viable fetuses and immune cells in the spleen, MLNs, PPs and blood was performed using Prism software version 9 (GraphPad Software, San Diego, USA). First, outliers were removed by means of the ROUT test (Q = 1%) (only for immune data), whereafter we tested for normality using the Kolmogorov-Smirnov test. When data were not normally distributed, data were log-transformed before analysis (data that were log-transformed before the analysis were indicated with a “#” in the graphs). In all graphs, individual values and median were displayed. One-way ANOVA was used to evaluate whether there were differences between the 3 groups (lean, obese and switch). Post-testing was performed with the Šídák’s multiple comparisons test. Since we were interested in the effect of obesity and the diet switch, post-tests were done on selected groups. For the effect of obesity, we compared obese vs. lean mice (differences indicated by “a” in the graphs), whereas we compared switch vs. obese mice (differences indicated by “b” in the graphs) to study the effect of the diet switch. Data were considered significantly different when p < 0.05.

Statistical analysis on maternal gut microbiota composition was performed using Past4 hammer (46). The PERMANOVA test was used to evaluate whether there were differences between groups, and the Shannon index was used to determine the alpha-diversity. Prism software version 9 was used to determine differences in bacterial phyla and bacterial genera (when PERMANOVA, p < 0.05) and in Shannon index between the groups by means of the Kruskal-Wallis test followed by Dunn’s multiple comparisons test (obese before pregnancy vs. lean before pregnancy, obese E7 vs. lean E7, obese E14 versus vs. E14, obese E18 vs. lean E18, switch E7 vs. obese E7, switch E14 vs. obese E14 and switch E18 vs. obese E18). Data were considered significantly different when p < 0.05. In all graphs, individual values and median were displayed.

For evaluating correlations between immune cells in the spleen, MLNs, PPs and blood and bacterial genera or fetal/placental weight, SPSS was used to determine Spearman’s rank correlation coefficients. We used immune subsets and bacterial genera that were different between obese vs. lean mice and between switch vs. obese mice at E18 pregnancy. Heatmaps were produced using CLUSTVIS (47), with cluster analysis for immune cells using Euclidean distance and Ward’s linkage.

## 3 Results

### 3.1 Th1 and Th17 cell subsets were increased in the peripheral and intestinal immune system of obese mice, and switching diet caused a decrease in Th2 cells

**Figure 2:**
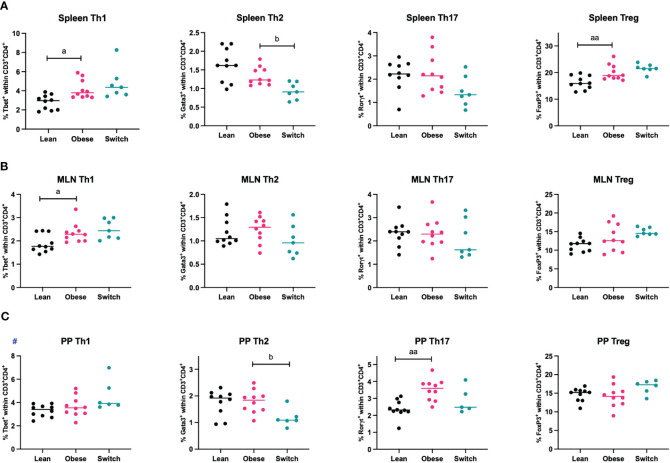
Frequencies of T helper 1 cells (Th1), T helper 2 cells (Th2), T helper 17 cells (Th17) and regulatory T cells (Treg) in the **(A)** spleen, **(B)** mesenteric lymph nodes (MLN) and **(C)** Peyer’s patches (PP) of lean, obese and switch mice at day 18 of pregnancy. One-way ANOVA followed by Šídák’s multiple comparisons test, significance levels: a = difference between obese and lean mice (a p < 0.05, aa p < 0.01), b = difference between obese and switch mice (b p < 0.05). # = log-transformed data. Lean mice: n = 10, obese mice: n = 10, switch mice: n = 5-7.

During healthy pregnancy, a proper balance between T helper cell subsets (Th1, Th2, Th17, Treg) is essential to promote maternal tolerance to the fetus (7–9). To investigate if maternal obesity affects T helper subsets in the peripheral circulation and intestinal immune system, mice were sacrificed at E18 of pregnancy, whereafter T helper cell subsets were quantified in the peripheral circulation (spleen) and intestinal immune system (MLN and PP) (**Figure 2**).

Th1 cell percentages were increased in the spleen (p < 0.05) and MLN (p < 0.05), but not in PP, of obese mice compared to lean mice. No obesity effect was observed for the percentage of Th2 cells in any of these tissues. Only in the PP of obese mice, the percentage of Th17 cells was increased (p < 0.01). Treg cell percentages were increased in the spleen (p < 0.01), but not in the MLN and PP. Th2 cell percentages were influenced by the diet switch. In the spleen (p < 0.05) and PP (p < 0.05) of switch mice, the percentage of Th2 cells was decreased as compared to obese mice.

There was no effect of obesity nor the diet switch on the percentages of cytotoxic T cells (CD3^+^CD8^+^) or T helper (CD3^+^CD4^+^) (**Supplementary Figure 6**).

### 3.2 Obesity induced enhanced production of proinflammatory cytokines by splenic T helper cells which was not ameliorated by switching diet

**Figure 3:**
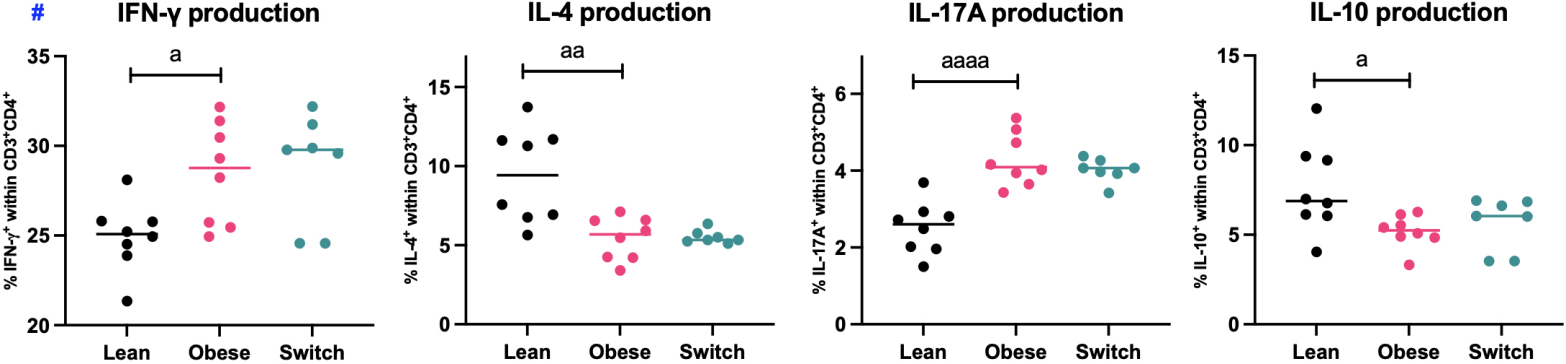
Frequencies of IFN-γ, IL-4, IL-17A and IL-10 producing splenic T helper cells (CD3^+^CD4^+^) of lean, obese and switch mice at day 18 of pregnancy. One-way ANOVA followed by Šídák’s multiple comparisons test, significance levels: a = difference between obese and lean mice (a p < 0.05, aa p < 0.01, aaaa p < 0.0001). # = log-transformed data. Lean mice: n = 8, obese mice: n = 8 and switch mice: n = 7.

As we found phenotypical differences in the distribution of splenic T helper cell subsets during maternal obesity, we also investigated functional characteristics of splenic T helper cells (CD3^+^CD4^+^) by the quantification of IFN-γ (Th1), IL-4 (Th2), IL-17A (Th17) and IL-10 (Treg) production upon stimulation of Th cells with PMA and ionomycin for four hours (**Figure 3**).

In obese pregnant mice, the percentages of IFN-γ (p < 0.05) and IL-17A (p < 0.0001) producing T helper cells were increased, while the percentages of IL-4 (p < 0.01) and IL-10 (p < 0.05) producing T helper cells were decreased as compared to controls. No differences were observed between switch mice and obese mice.

### 3.3 Monocyte subsets were altered in obese mice, switching diet reverted this effect and impacted the activation status of monocytes

**Figure 4:**
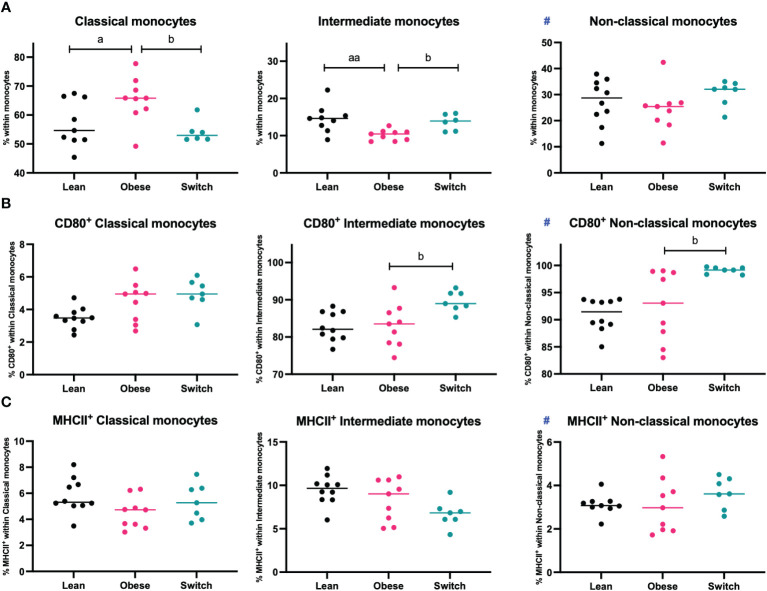
Frequencies of **(A)** classical monocytes, intermediate monocytes, and non-classical monocytes, **(B)** CD80^+^ classical, intermediate, and non-classical monocytes and **(C)** MHCII^+^ classical, intermediate, and non-classical monocytes in the blood of lean, obese and switch mice at day 18 of pregnancy. Data are presented as individual values and median. One-way ANOVA followed by Šídák’s multiple comparisons test, a = difference between obese and lean mice (a p < 0.05, aa p < 0.01), b = difference between obese and switch mice (b p < 0.05). # = data were log-transformed before statistical analysis. Lean mice: n = 8/9, obese mice: n = 9 and switch mice: n = 6/7.

Monocytes are divided into three populations and have distinct functions. Classical monocytes are primed for phagocytosis and exhibit the most pro-inflammatory phenotype, intermediate monocytes are specialized in antigen presentation and non-classical monocytes are mostly involved in tissue remodeling (13). During uncomplicated pregnancy, classical monocytes decrease, while non-classical monocytes increase, and the activation status of monocytes is enhanced to prevent rejection of the fetus (11, 12). Since maternal tolerance to the fetus also depends on monocyte subsets and their activation status, we hypothesized that maternal obesity might also affect these parameters. To investigate this, monocyte subsets and activation status (as measured by CD80 and MHCII) were determined in the maternal blood at E18 of pregnancy (**Figure 4**).

Intermediate monocytes were decreased (p < 0.01), while classical monocytes were increased (p < 0.05) in the blood of obese mice compared to lean mice. No differences were observed for non-classical monocytes, or the expression of CD80 or MHCII on the monocyte subsets. Monocyte subsets were also influenced by the diet switch. Intermediate monocytes were increased (p < 0.05), while classical monocytes were decreased (p < 0.05) in the blood of switch mice as compared to obese mice. Monocyte activation was most strongly influenced by the diet switch: an increased expression of CD80 was found in the blood of switch mice, on both non-classical (p < 0.01) and intermediate (p < 0.05) monocytes, as compared to obese pregnant mice.

### 3.4 Obesity and diet independently alter the maternal gut microbiota composition in pregnant mice

Various studies have shown that the immune response in general is regulated by the gut microbiome (26, 27), while other studies have shown that maternal obesity is associated with microbial gut dysbiosis (30–32). Therefore, we initially investigated whether the longitudinal collected gut microbiota composition of obese mice differed from the gut microbiota composition of lean mice. Differences in microbiota diversity and relative abundance were determined in fecal samples with 16S rRNA sequencing.

**Figure 5:**
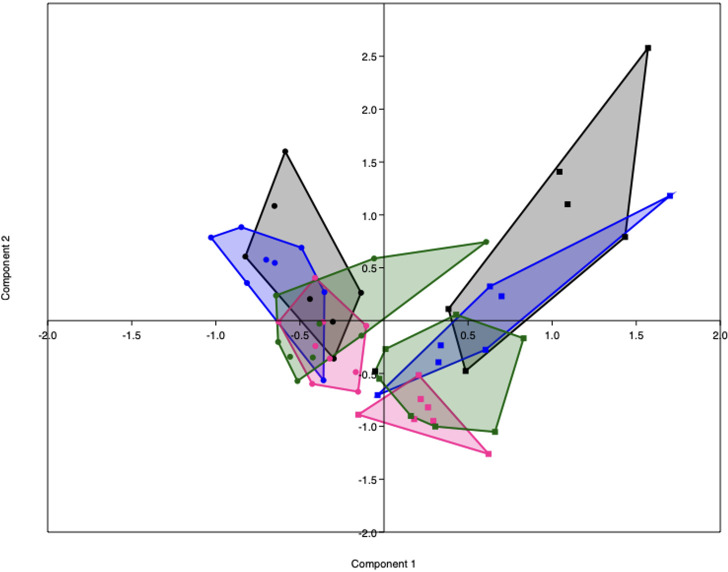
PCA plot representing the gut microbiota composition of lean (dots) and obese (squares) mice before (black) and during pregnancy at day 7 (blue), day 14 (pink) and day 18 (green). Lean mice before: n = 7, lean mice E7: n = 8, lean mice E14: n = 9, lean mice E18: n = 9, obese mice before: n =6, obese mice E7: n = 7, obese mice E14: n = n = 7 and obese mice E18: n = 8.

PCA analysis revealed that the gut microbiota of obese mice differed from the gut microbiota of lean mice, both before (PERMANOVA, p < 0.001) and during pregnancy on E7 (PERMANOVA, p < 0.0001), E14 (PERMANOVA, p < 0.001) and E18 (PERMANOVA, p < 0.0001) (**Figure 5**). No differences in alpha diversity, as measured by the Shannon index, were observed between obese and lean mice (**Supplementary Figure 7**).

As immune responses were differently influenced by obesity and the diet switch, we also investigated the effect of the diet switch on the gut microbiota (**Supplementary Table 4**). Before the diet switch on E7 of pregnancy, the gut microbiota composition of the switch group did not significantly differ from the obese group (PERMANOVA, p > 0.05). After the diet switch, the gut microbiota of switch mice differed from obese mice, both at E14 (PERMANOVA, p < 0.05) and E18 (PERMANOVA, p < 0.01) of pregnancy.

**Figure 6:**
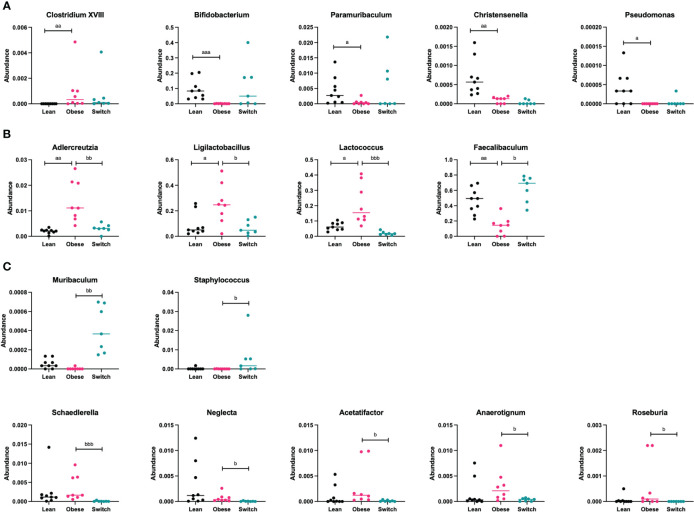
**(A)** Bacterial genera that were mainly altered by the obese state and not the diet type (*Clostridium XVIII*, *Bifidobacterium*, *Paramuribaculum*, *Christensenella*, *Pseudomonas*) ay day 18 of pregnancy. **(B)** Bacterial genera that were mainly altered by the diet type and not the obese state (*Adlercreutzia*, *Ligilactobacillus*, *Lactococcus*, *Faecalibaculum*) at day 18 of pregnancy. **(C)** Bacterial genera that were mainly altered by the event of the diet switch, rather than the obese state or the diet type (*Muribaculum*, *Staphylococcus*, *Schaedlerella*, *Neglecta*, *Acetatifactor*, *Anaerotignum*, *Roseburia*) at day 18 of pregnancy. Data are presented as individual values and median. Kruskal-Wallis-test followed by Dunn’s multiple comparisons test (statistical differences were taken from **Supplementary Tables 4**, **5**, in which we respectively compared obese with lean and obese with switch mice at different pregnancy days). a = difference between obese and lean mice (a p < 0.05, aa p < 0.01, aaa p < 0.001), b = difference between obese and switch mice (b p < 0.05, bb p < 0.01, bbb p < 0.001). Lean mice: n = 9, obese mice: n = 8 and switch mice: n =7.

Next, bacterial phyla and genera were compared between obese and lean mice and obese and switch mice. **Supplementary Table 5** gives a complete overview of all the significantly different phyla and genera before and during pregnancy at E7, 14 and 18 between obese and lean mice. While **Supplementary Table 6** gives a complete overview of all the significantly different genera at E14 and 18 between obese and switch mice. We highlight the most prominent bacterial genera that significantly differed between obese and lean and obese and switch mice at E18 of pregnancy (**Figure 6**).

Specific bacterial genera were mainly altered by the obese state and not the diet type, as the genera *Clostridium XVIII* (p < 0.001; increased), *Bifidobacterium* (p < 0.001; decreased), *Paramuribaculum* (p < 0.05; decreased), *Christensenella* (p < 0.01; decreased) and *Pseudomonas* (p < 0.05; decreased) were different in obese versus lean mice, but not in switch versus obese mice (**Figure 6A**).

Several bacterial genera were mainly altered by the diet type and not the obese state, as the genera *Adlercreutzia* (p < 0.01), *Ligilactobacillus* (p < 0.05) and *Lactococcus* (p < 0.001), which were more abundant in obese mice than in lean mice, were decreased in switch versus obese mice. While the genus *Faecalibaculum* (p < 0.05), which was less abundant in obese mice than in lean mice, was increased in switch versus obese mice (**Figure 6B**).

Other bacterial genera were mainly altered by the event of the diet switch, rather than the obese state or the diet type, as several genera that did not differ between obese and lean mice, including *Muribaculum* (p < 0.01; increased), *Staphylococcus* (p < 0.05; increased), *Schaedlerella* (p < 0.001; decreased), *Neglecta* (p < 0.05; decreased), *Acetatifactor* (p < 0.05; decreased), *Anaerotignum* (p < 0.05; decreased) and *Roseburia* (p < 0.05; decreased) were different in switch versus obese mice (**Figure 6C**).

### 3.5 Correlations between the gut microbiota and maternal immune cell subsets that differed between obese and lean and between obese and switch mice

**Figure 7:**
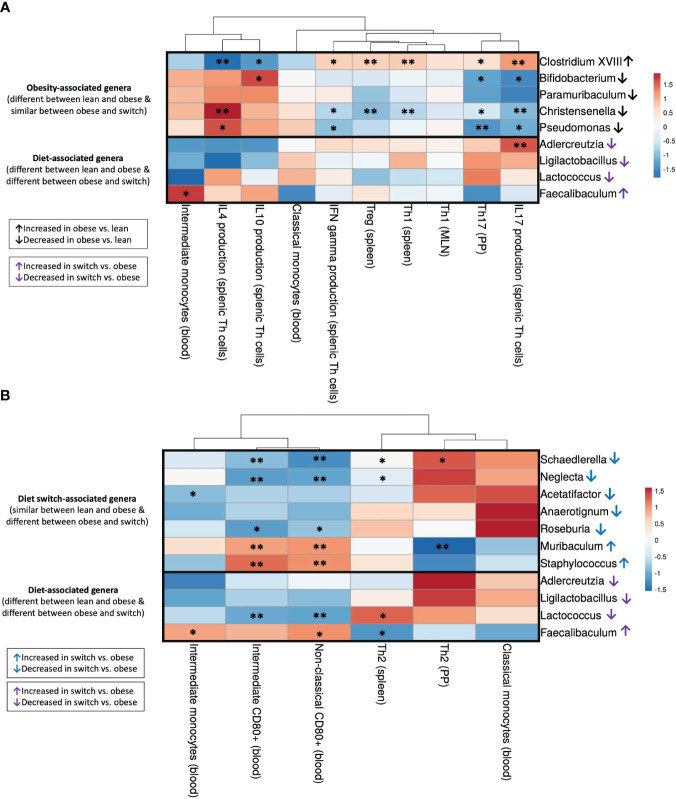
Correlations between gut microbiota and immune cell subsets that differed between **(A)** obese and lean mice (Th1 (spleen), Treg (spleen), Th1 (MLN), Th17 (PP), IFN-γ/IL-4/IL-17A/IL-10 production by splenic Th cells, classical/intermediate monocytes) and **(B)** obese and switch mice (Th2 (spleen), Th2 (PP), classical/intermediate monocytes, CD80^+^ intermediate/non-classical monocytes) at day 18 of pregnancy. Figures show heatmaps of Spearman’s correlation coefficients after individual correlation of immune cell populations (x-axis) and bacterial genera (y-axis). Bacterial genera were classified into 3 groups: (1) bacterial genera that were changed by the obese state (*Clostridium XVIII*, *Bifidobacterium*, *Paramuribaculum*, *Christensenella*, *Pseudomonas*), (2) bacterial genera that were changed by the type of diet (*Adlercreutzia*, *Ligilactobacillus*, *Lactococcus*, *Faecalibaculum*), and (3) bacterial genera that were changed by the event of the diet switch (*Schaedlerella*, *Neglecta*, *Acetatifactor*, *Anaerotignum*, *Roseburia*, *Muribaculum*, *Staphylococcus*). Lean mice: n = 9, obese mice: n = 8 and switch mice n = 7. * p < 0.05, ** p < 0.01).

Next, to gain insight into the relationship between immune cell changes and the gut microbiota, we correlated individual immune cell data and microbiota abundance of the same mouse (obese, lean and switch) at E18 of pregnancy. For immune cells, we included subsets that were significantly different between obese versus lean mice (Th1 (spleen), Treg (spleen), Th1 (MLN), Th17 (PP), IFN-γ/IL-4/IL-17A/IL-10 production by splenic Th cells, classical/intermediate monocytes) (**Figure 7A**) and obese versus switch mice (Th2 (spleen), Th2 (PP), classical/intermediate monocytes, CD80^+^ intermediate/non-classical monocytes) (**Figure 7B**). Bacterial genera were classified into 3 groups (1): bacterial genera that were changed by the obese state (*Clostridium XVIII*, *Bifidobacterium*, *Paramuribaculum*, *Christensenella*, *Pseudomonas*), (2) bacterial genera that were changed by the type of diet (*Adlercreutzia*, *Ligilactobacillus*, *Lactococcus*, *Faecalibaculum*), and (3) bacterial genera that were changed by the event of the diet switch (*Schaedlerella*, *Neglecta*, *Acetatifactor*, *Anaerotignum*, *Roseburia*, *Muribaculum*, *Staphylococcus*). Spearman’s rank correlation coefficients are shown in heatmaps.

#### 3.5.1 Significant correlations between the obesity-associated gut microbiota and immune cell subsets that differed between obese and lean mice

The bacterial genus *Clostridium XVIII*, which was increased by the obese state, negatively correlated with IL-4 and IL-10 production by splenic Th cells and positively correlated with IFN-γ and IL-17A production by splenic Th cells and percentages of Th1 and Treg cells in the spleen and Th17 cells in the PPs (**Figure 7A**). The opposite effect was observed for the bacterial genera *Bifidobacterium*, *Christensenella* and *Pseudomonas*, which were decreased by the obese state; most of these genera were positively correlated with IL-4 and IL-10 production by splenic Th cells and negatively correlated with IFN-γ and IL-17A production by splenic Th cells and percentages of Th1 and Treg cells in the spleen and Th17 cells in the PPs.

#### 3.5.2 Significant correlations between the diet-associated gut microbiota and immune cell subsets that differed between obese and lean and obese and switch mice

The genus *Adlercreutzia*, which decreased after the switch from the HFD to the LFD, positively correlated with IL-17A production by splenic Th cells (**Figure 7A**). *Lactococcus*, another genus that was decreased after the switch from the HFD to the LFD, positively correlated with the percentage of splenic Th2 cells and negatively correlated with CD80 expression on intermediate and non-classical monocytes in the blood (**Figure 7B**). On the contrary, the genus *Faecalibaculum*, which increased after the switch from the HFD to the LFD, positively correlated with the percentage of intermediate monocytes and CD80 expression on non-classical monocytes in the blood and negatively correlated with the percentage of splenic Th2 cells (**Figures 7A, B**).

#### 3.5.3 Significant correlations between the diet switch-associated gut microbiota and immune cell subsets that differed between obese and switch mice

Most of the genera which were decreased by the event of the diet switch, including *Schaedlerella*, *Neglecta*, *Acetatifactor* and *Roseburia*, positively correlated with the percentage of Th2 cells in the spleen and PPs, while these genera negatively correlated with the percentage of intermediate monocytes and CD80 expression on intermediate and non-classical monocytes (**Figure 7B**). The opposite effect was observed for the bacterial genera *Muribaculum* and *Staphylococcus*, which were increased by the event of the diet switch; these genera negatively correlated with the percentage of Th2 cells in the PP, while these genera positively correlated with CD80 expression on intermediate and non-classical monocytes.

### 3.6 Fetal weight is decreased in obese mice and switching diet induces a loss of placental weight

**Figure 8:**
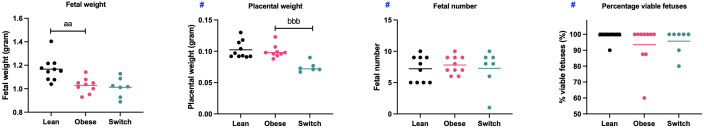
Mean fetal weight, placental weight, fetal number and percentage of viable fetuses per dam obtained from lean, obese and switch mice. One-way ANOVA followed by Šídák’s multiple comparisons test, significance levels: a = difference between obese and lean mice (aa p < 0.01), b = difference between obese and switch mice (bbb p < 0.01). # = log-transformed data. Lean mice: n = 10, obese mice: n = 9/10 and switch mice: n = 6/7.

Mean fetal weight, placental weight, the total number of fetuses and the percentage of viable fetuses per dam were taken as a measure for pregnancy outcome and were determined at E18 of pregnancy (**Figure 8**).

In obese mice, fetal weight was lower (1.03 gram ± 0.02) than in lean mice (1.17 gram ± 0.03) (p < 0.01), with no effects on placental weight, fetal number and the percentage of viable fetuses. In the switch mice, placental weight (0.075 grams ± 0.003) was lower compared to the obese mice (0.099 grams ± 0.003) (p < 0.001), with no differences observed in the other parameters.

### 3.7 Correlations between fetal and placental weight and maternal immune cells that are impacted by obesity, the diet or the diet switch

**Figure 9:**
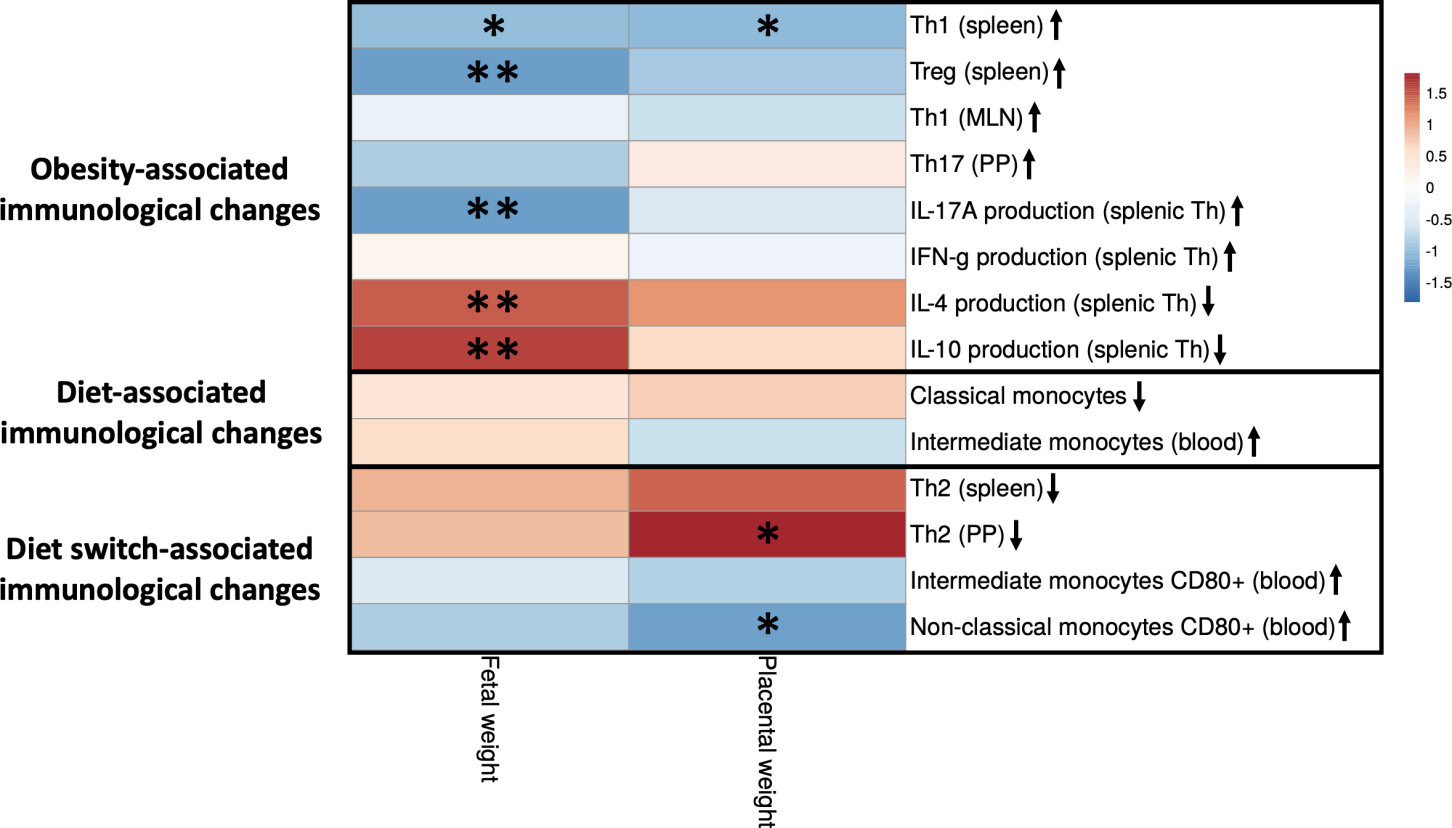
Correlations between fetal and placental weight and maternal immune cells that are impacted by obesity, the diet or the diet switch at day 18 of pregnancy. The figure shows a heatmap of Spearman’s correlation coefficients after individual correlation of immune cell populations (x-axis) and fetal/placental weight (y-axis) at day 18 of pregnancy. Maternal immunological changes were classified into 3 groups: (1) obesity-associated immunological changes that were only observed between obese and lean mice but not between obese and switch mice, (2) diet-associated immunological changes that were observed between obese and lean and between obese and switch mice, (3) diet switch-associated immunological changes that were observed between obese and switch mice but not between obese and lean mice. Lean mice: n = 9, obese mice: n = 8 and switch mice n = 7. * p < 0.05, ** p < 0.01.

Next, to gain insight into the relationship between the observed immune cell changes and the decreased fetal and placental weight, we correlated individual immune cell data and fetal/placental weight of the same mouse (obese, lean and switch) at E18 of pregnancy. For immune cells, we included subsets that were significantly different between obese and lean and/or obese and switch mice (**Figure 9**).

Maternal immunological changes were classified into 3 groups: (1) obesity-associated immunological changes that were only observed between obese and lean mice but not between obese and switch mice (increased percentages of Th1 (spleen), Treg (spleen), Th1 (MLN), Th17 (PP) and IFN-γ/IL-17A producing splenic Th cells and decreased percentages of IL-4/IL-10 producing splenic Th cells), (2) diet-associated immunological changes that were observed between obese and lean and between obese and switch mice (increased percentage of classical and decreased percentage of intermediate monocytes when fed the HFD), (3) diet switch-associated immunological changes that were observed between obese and switch mice but not between obese and lean mice (decreased percentage of Th2 cells (spleen and PP) and increased expression of CD80 on intermediate and non-classical monocytes).

Several significant correlations were found between obesity-associated immunological changes and fetal weight. The production of IL-4 and IL-10 by splenic Th cells was positively correlated with fetal weight. While percentages of Th1 and Treg cells in the spleen and the production of IL-17A by splenic Th cells were negatively correlated with fetal weight. Diet switch-associated immunological changes mostly significantly correlated with placental weight, as the percentage of Th2 cells in the PP was positively correlated with placental weight. CD80 expression on non-classical monocytes in the blood was negatively correlated with placental weight. Also, the percentage of splenic Th1 cells correlated negatively with placental weight.

## 4 Discussion

**Figure 10:**
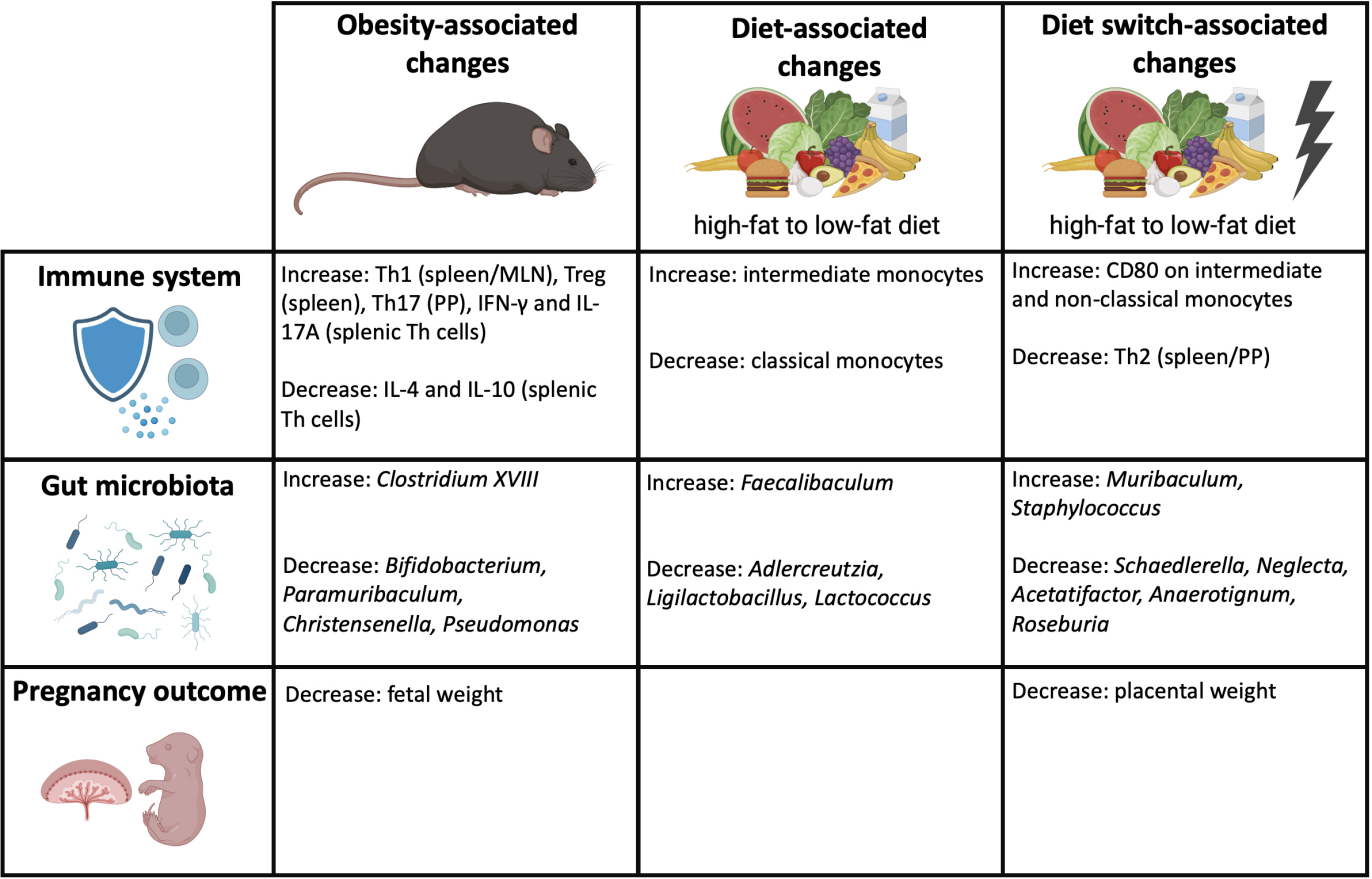
Overview of the obesity-associated, diet-associated and diet switch-associated changes that were observed in this study. Created with BioRender.com.

It remains unclear how the state of obesity affects pregnancy. We hypothesized that maternal obesity induces anomalies in immunity, which can be mediated by obesity-induced dysbiosis of the maternal gut microbiota. Our study confirms our hypothesis by demonstrating that maternal obesity causes derangements in the maternal peripheral and intestinal immune responses, which are accompanied by alterations of specific bacterial genera within the gut microbiota composition and decreased fetal weight at the end of pregnancy. Importantly, besides obesity, our data show that dietary patterns can independently impact the gut microbiota composition, maternal immune responses and pregnancy outcomes. An overview of the obesity-associated changes, diet-associated changes and diet switch-associated changes are shown in **Figure 10**.

Our study demonstrates that maternal obesity influences peripheral immune responses by influencing the Th1/Th2/Th17/Treg axis and monocyte subsets in obese mice. We found enhanced splenic Th1 cells in obese mice with an increased IFN-γ production upon stimulation, while despite an increased percentage of splenic Treg cells, IL-10 production by splenic Th cells was diminished. These findings suggest a reduced Treg function in obese mice which is compensated by a larger Treg cell pool. Most of our findings on the effects of obesity on the immune system are in line with a previous human study (24), and also appear to apply to the general non-pregnant population (17, 19).

Our study not only demonstrates that maternal obesity has a large influence on the peripheral immune response but also induces anomalies in the intestinal adaptive immune system (MLN and PP). To the best of our knowledge, we are the first to investigate Th subsets within the MLN and PP in obese pregnant mice. Previously, we have shown that Th cell subsets change in both the peripheral and intestinal immune system during healthy pregnancy in lean mice (27). This suggest that the maternal gut microbiota affects the intestinal immune response, which in turn affects the maternal peripheral immune response. In the MLN and PP of obese mice, we observed increased percentages of respectively Th1 and Th17 cells, which are considered proinflammatory responses. Upregulation of gut Th1 and Th17 cells is associated with diseases such as inflammatory bowel and Crohn’s disease (48, 49). On the other hand, upregulation of gut Th17 cells could also be perceived as a counterregulatory response to the obesity-induced dysbiosis since it is a potent producer of IL-22 (50). IL-22 is considered a maturation factor for gut epithelial cells that induces expression of bacterial adhesion molecules such as FUT-2, facilitating adhesion of commensals (51, 52).

Maternal obesity also had a strong impact on the gut microbiota composition. The increased abundance of the genus *Clostridium XVIII*, and decreased abundances of the genera *Bifidobacterium*, *Paramuribaculum*, *Christensenella* and *Pseudomonas* in obese mice, but not in switch mice suggest an association with the obese state but not the consumed diet type. Our findings also corroborate the findings in humans where also an increase of *Clostridium* was found during (maternal) obesity as well as a decrease of genera such as *Bifidobacterium*, *Christensenella* and *Pseudomonas* (31, 53–55). Importantly, our data demonstrate many correlations between obesity-induced anomalies in Th cell subsets and obesity-associated bacterial genera. This suggest that specific genera are involved in the modulation of the peripheral and intestinal immune response during maternal obesity. Various studies have shown that certain bacterial genera influence Th cell differentiation, either via direct contact with immune cells in the gut or via the release of microbial products, such as SCFAs and bile acids, corroborating our findings (28, 56–62). For instance, specific *Bifidobacterium* strains are known to reduce Th17 cells in the gut and can inhibit IL-17A production by murine splenocytes (56–58), while the genus *Clostridium XVIII* is a potent inducer of Treg cells (59). Furthermore, *Bifidobacterium* and *Christensenella* are important producers of SCFAs, which promote the production of IL-10 by Treg cells (60). In addition, these genera are also involved in the formation of secondary bile acids, which modulate the differentiation of Treg cells and inhibit Th17 differentiation (61, 62). Also, several *Bifidobacterium* and *Christensenella* species have already shown therapeutic anti-obesity potential in non-pregnant (obese) mouse models by decreasing obesity-associated inflammation (63–66).

Correlation analysis showed that the obesity-induced changes in immunity and therewith the gut microbiota had a clear negative effect on pregnancy outcome such as on fetal weight. A possible explanation for the impacted fetal weight is that obesity induced by a HFD impairs the function of the placenta causing fetal growth restriction (67–69). This was also found in other studies in which rodents were fed a HFD during the periconceptional period (70–73). Our data suggest that the observed changes in the maternal gut microbiota composition and immune system might be partly involved in this potential causative pathway. Both we and others support this argumentation by showing that maternal microbiota support placental development in mice (74), and that antibiotic-induced dysbiosis of the maternal microbiota restricts placental and fetal growth (75) and impairs fetoplacental vascularization (76). The lower abundance of *Bifidobacterium* in obese pregnant mice may be a responsible microbial genus as this genus promotes placental morphogenesis, nutrient transport and fetal growth in mice (74). The dysbiosis-induced increase in Th1 response during obese pregnancy is also known to be responsible for fetal growth restriction (14, 15).

Several bacterial genera were altered by the diet rather than by the obese state. Correlation analysis showed that diet-associated-genera such as *Adlercreutzia*, *Ligilactobacillus*, *Lactococcus and Faecalibaculum* also impact maternal immune responses, independent of the obese state, with the strongest effect on monocytes. In our study, the dietary pattern enhanced the classical monocytes and decreased the intermediate monocytes. Our data therefore suggest that diet type is mainly responsible for the monocyte subset anomalies that were observed in obese mice. Other studies support our findings, as it was shown that monocyte subsets were extremely responsive to changes in plasma lipid profiles (77, 78), and that HFDs caused remodeling of bone marrow adipocytes that disrupted the balance of monocytes in the circulation (79). Also, a human study found that intermediate monocytes were diminished with increasing maternal BMI at the beginning of the third trimester (24). Here we show that this might be induced by diet rather than by the obese status of the pregnant individual.

Our data suggest that switching diet from an HFD to an LFD during early pregnancy may not be advisable, since this induces additional changes in the maternal gut microbiota composition and the immune response that seem to negatively impact pregnancy outcome. As a consequence of the diet switch, we found increased abundances of *Muribaculum* and *Staphylococcus* and decreased abundances of *Schaedlerella*, *Neglecta*, *Acetatifactor*, *Anaerotignum* and *Roseburia* as compared to obese mice. Various of these genera correlated with the decreased Th2 response and increased CD80 expression on intermediate and classical monocytes, suggesting that the diet switch-associated genera are mainly responsible for these immunological changes. A decreased Th2 response and increased monocyte activation (as indicated by increased CD80 expression) are associated with negative pregnancy outcome (11, 80).

The effect of the diet switch on the immunological changes might be explained by the fact that change of diet may impact gut permeability (81), inducing a change in the intestinal immune response (36), which may affect the peripheral immune response. A remarkable finding was that the placental weight was also severely impacted by the diet switch. Based on our data we propose that the changes in the diet switch-associated maternal gut microbiota composition and associated changes in immune responses may have affected placental growth as discussed above. However, also the change in nutrition may be involved, as the murine placenta is finalized around E12 of pregnancy (82) and placental development is highly dependent on maternal nutrient intake during early pregnancy (83). Switching from a HFD to a LFD at E7 of pregnancy most likely also causes major shifts in circulating lipid profiles inducing changes in metabolic processes that might ultimately affect placental development (81, 84).

## 5 Concluding remarks

Collectively, our data suggest that maternal obesity as such induced changes in specific bacterial genera within the maternal gut microbiota and anomalies in the peripheral and intestinal maternal immune response and a lowered fetal weight in C57/BL6 mice. Importantly, we also show that it is not only maternal obesity as such but also the diet type that impacts maternal immunity, the gut microbiota composition and fetal growth. However, our data also suggests that switching diet from an HFD to an LFD during early pregnancy may not be advisable, since this event set of additional changes in the maternal immune response and gut microbiota composition that negatively impacted pregnancy outcome. As switching diet might not help, we suggest that correction of obesity-induced gut dysbiosis, for instance by pre- or probiotic treatment, may ameliorate these anomalies during pregnancy.

## Data availability statement

The data 16S rRNA sequencing data presented in this study are deposited in the NCBI Sequence Read Archive (SRA) repository, accession number PRJNA1131383.

## Ethics statement

The animal study was approved by the Central Committee on Animal Experimentation in the Netherlands. The study was conducted in accordance with the local legislation and institutional requirements.

## Author contributions

LWe: Conceptualization, Formal analysis, Investigation, Methodology, Project administration, Writing – original draft. SS: Conceptualization, Funding acquisition, Methodology, Resources, Writing – review & editing. NS: Conceptualization, Methodology, Writing – review & editing. AL: Investigation, Writing – review & editing. LL: Formal analysis, Writing – review & editing. LWa: Investigation, Writing – review & editing. HH: Investigation, Writing – review & editing, Formal analysis. RS: Conceptualization, Funding acquisition, Methodology, Resources, Writing – review & editing. MF: Conceptualization, Funding acquisition, Investigation, Methodology, Resources, Supervision, Writing – review & editing.
