# Supplementary material for: Obesity and diet independently affect maternal immunity, maternal gut microbiota and pregnancy outcome in mice
Source: Front Immunol. 2024 Jul 12;15:1376583. doi: 10.3389/fimmu.2024.1376583 (PMC11272480; doi:10.3389/fimmu.2024.1376583)
Supplement: Supplementary file 1 [file DataSheet_1.docx]

**Obesity and Diet Independently Affect Maternal Immunity, Maternal Gut Microbiota and Pregnancy Outcome in Mice**

Lieske Wekema, Sam Schoenmakers, Nicole Schenkelaars, Anne Laskewitz, Lei Liu, Lisa Walters, Hermie J.M. Harmsen, Régine P.M. Steegers-Theunissen, Marijke M. Faas

Supplementary Material

**
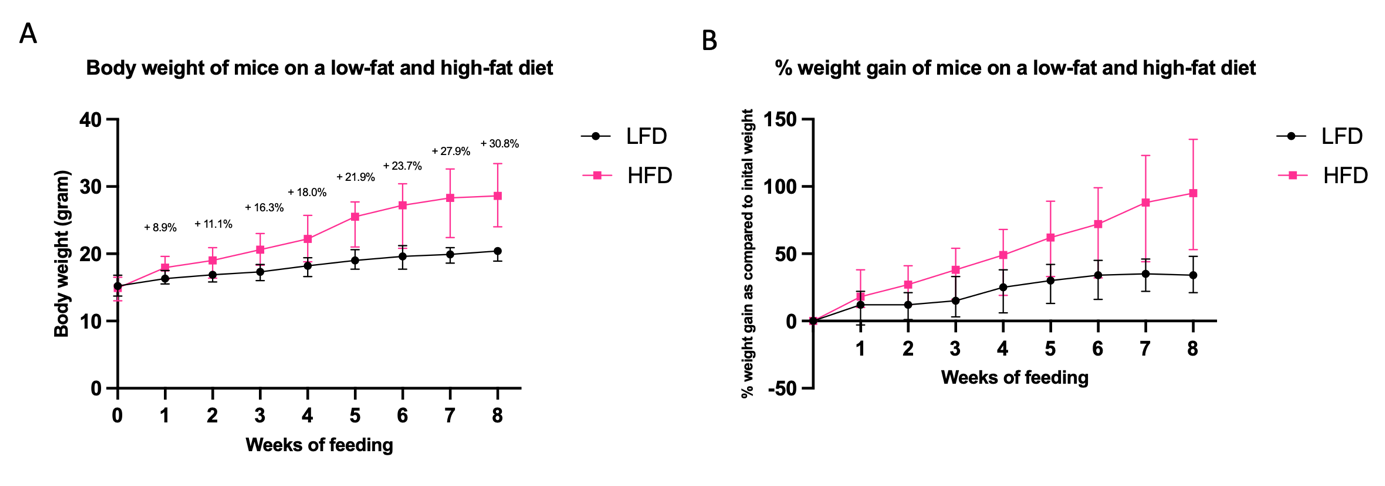
**

**Supplementary Figure 1.** **(A)** Growth curves of female C57BL/6 mice fed a high-fat diet (HFD) (n = 17) or low-fat diet (LFD) (n = 10). Data are presented as median ± range. Per week the percentage of weight gain of the HFD mice is depicted as compared to the LFD mice. After 8 weeks of feeding, the body weight of the mice on the HFD (28.81 gram ± 0.82) exceeded the bodyweight of the mice on the LFD (19.94 gram ± 0.25) by 30%. **(B)** Percentage weight gain of female C57BL/6 mice fed a high-fat diet (HFD) (n = 17) or low-fat diet (LFD) (n = 10) as compared to the initial body weight at week 0. Data are presented as median ± range.


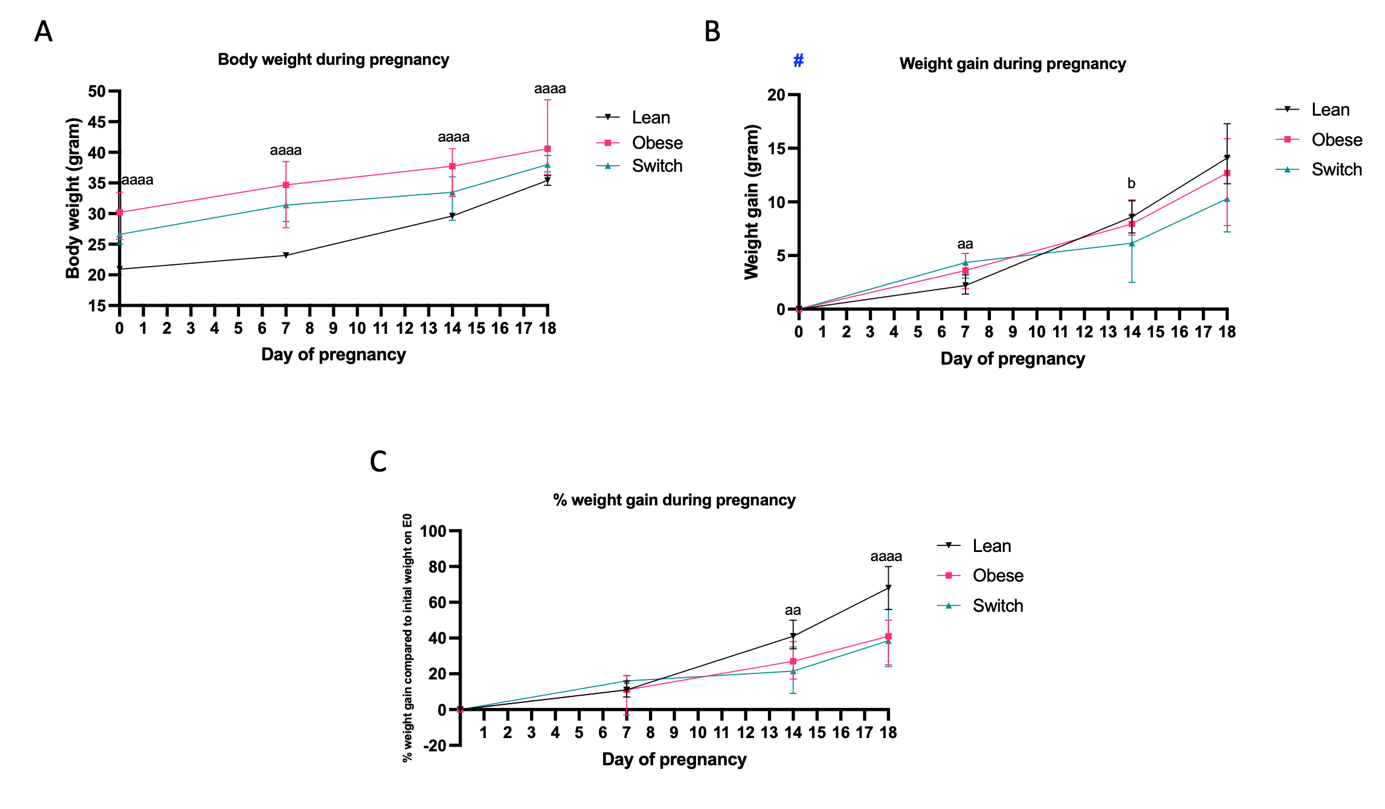


**Supplementary Figure 2.** **(A)** Body weight of lean (n =10), obese (n = 10) and switch (n = 7) C57BL/6 mice during pregnancy at day 0, 7, 14 and 18. Data are presented as median ± range. One-way ANOVA followed by Šídák's multiple comparisons test, a = difference between obese and lean mice (aaaa p < 0.0001). During pregnancy, the weight of the obese group remained significantly higher than the weight of the lean group at all days tested (p < 0.0001). No significant decrease in body weight was observed between the switch group and the obese group, indicating that the mice in the switch group remained obese. **(B)** Weight gain of lean (n = 10), obese (n = 10) and switch (n = 7) C57BL/6 mice during pregnancy at day 7, 14 and 18 as compared to day 0. Data are presented as median ± range. One-way ANOVA followed by Šídák's multiple comparisons test, a = difference between obese and lean mice (aa p < 0.01), b = difference between obese and switch mice (b p < 0.05). # = data were log-transformed before statistical analysis. **(C)** Percentage weight gain of lean (n = 10), obese (n = 10) and switch (n = 7) C57BL/6 mice during pregnancy at day 7, 14 and 18 as compared to day 0. Data are presented as median ± range. One-way ANOVA followed by Šídák's multiple comparisons test, a = difference between obese and lean mice (aa p < 0.01, aaaa p < 0.0001). At E14 (p < 0.01) and E18 (p < 0.0001) of pregnancy, the percentage of weight gain was significantly lower in the obese group as compared to the lean group.

**Supplementary Table 1.** Antibody mix for T helper cell staining

| **Antibody** | **Clone** | **Fluorochrome** | **Manufacturer** | **Cat.** | **Mix** | **Dilution** |
| --- | --- | --- | --- | --- | --- | --- |
| anti-CD3 | 17A2 | BV605 | Biolegend | 100237 | Extracellular | 1:25 |
| anti-CD4 | GK1.5 | PerCp-Cy5.5 | Biolegend | 100434 | Extracellular | 1:75 |
| anti-CD8 | 53-6.7 | PE-Cy7 | Biolegend | 100722 | Extracellular | 1:200 |
| anti-Tbet | 4B10 | BV421 | Biolegend | 644816 | Intracellular | 1:10 |
| anti-Rorγt | B2D | PE | Invitrogen | 12-6981-82 | Intracellular | 1:100 |
| anti-Gata3 | L50-823 | AF647 | BD Biosciences | 560068 | Intracellular | 1:100 |
| anti-FoxP3 | FJK-16s | FITC | Invitrogen | 11-5773-82 | Intracellular | 1:50 |

**Supplementary Table 2.** Antibody mix for cytokine producing splenic T helper cell staining

| **Antibody** | **Clone** | **Fluorochrome** | **Manufacturer** | **Cat.** | **Mix** | **Dilution** |
| --- | --- | --- | --- | --- | --- | --- |
| anti-CD3 | 17A2 | BV605 | Biolegend | 100237 | Extracellular | 1:25 |
| anti-CD4 | RM4-5 | V500 | BD Biosciences | 560782 | Extracellular | 1:100 |
| anti-CD8 | 53-6.7 | PerCp-Cy5.5 | Biolegend | 100734 | Extracellular | 1:50 |
| anti-IFNγ | XMG1.2 | PE | Biolegend | 505808 | Intracellular | 1:500 |
| anti-IL-4 | BVD6-24G2 | FITC | Invitrogen | 11-7042-82 | Intracellular | 1:400 |
| anti-IL-17A | TC11-18H10.1 | APC | Biolegend | 506916 | Intracellular | 1:500 |
| anti-IL-10 | JES5-16E3 | BV421 | Biolegend | 505022 | Intracellular | 1:250 |

**Supplementary Table 3.** Antibody mix for monocyte staining

| **Antibody** | **Clone** | **Fluorochrome** | **Manufacturer** | **Cat.** | **Mix** | **Dilution** |
| --- | --- | --- | --- | --- | --- | --- |
| anti-CD11b | M1/70 | PE | Biolegend | 101208 | Extracellular | 1:50 |
| anti-Ly6G | 1A8 | BV605 | BD Biosciences | 563005 | Extracellular | 1:25 |
| anti-CD115 | AFS98 | PE-Cy7 | Biolegend | 135525 | Extracellular | 1:60 |
| anti-CD43 | S11 | APC | Biolegend | 143208 | Extracellular | 1:100 |
| anti-Ly6C | HK1.4 | AF488 | Biolegend | 128022 | Extracellular | 1:200 |
| anti-CD80 | 16-10A1 | PB | Biolegend | 104724 | Extracellular | 1:25 |
| anti-MHCII | M5/114.15.2 | PerCp-Cy5.5 | Biolegend | 107626 | Extracellular | 1:200 |


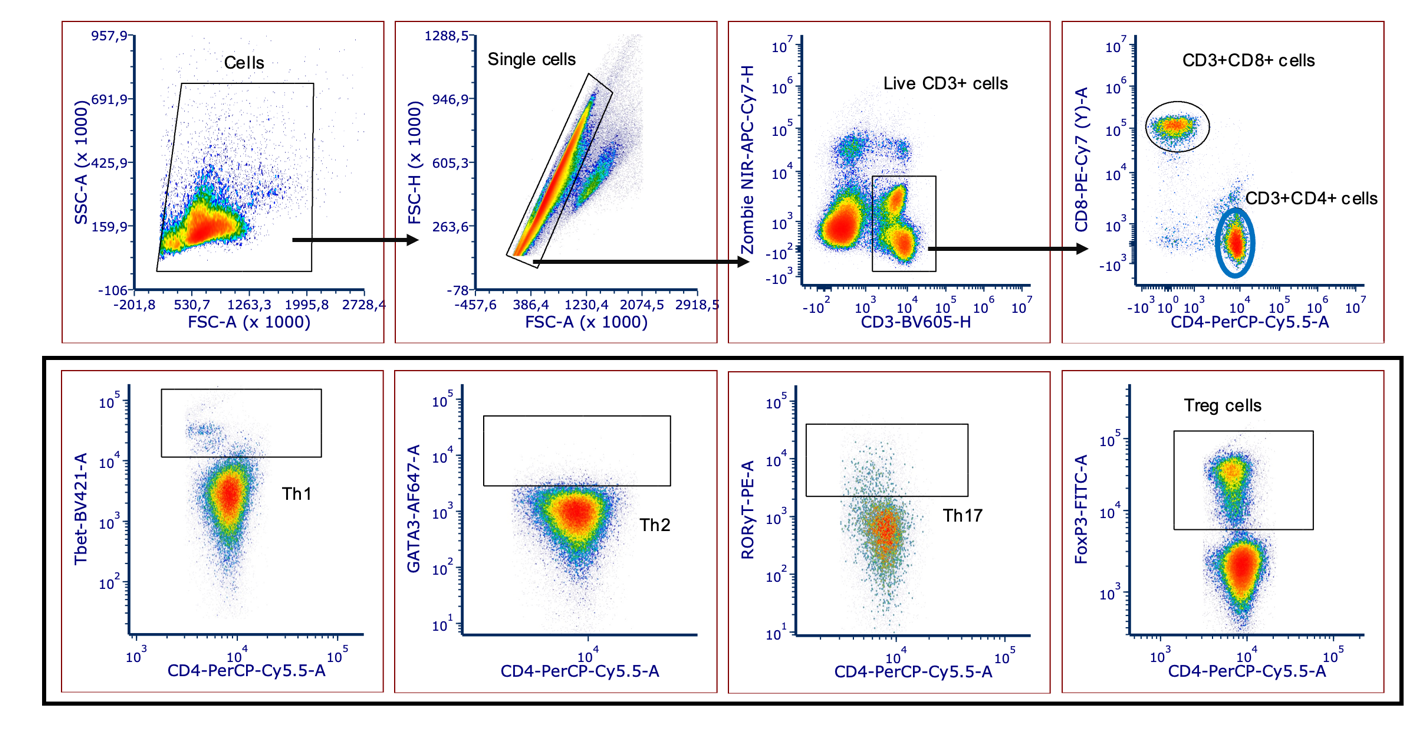


**Supplementary Figure 3.** Gating strategy for the determination of T helper cell subsets in the spleen, mesenteric lymph nodes and Peyer’s patches. Cells were selected based on their characteristic forward and side scatter properties. Next single cells were selected, whereafter live T cells (Zombie NIR^-^CD3^+^) were selected. T helper cells (CD3^+^CD4^+^) and cytotoxic T cells (CD3^+^CD8^+^) were selected within the live T cell population. The different subsets of T helper cells were selected by gating Tbet^+^ (Th1), Gata3^+^ (Th2), RORϒ^+^ (Th17) and FoxP3^+^ (regulatory T cell; Treg) T helper cells.


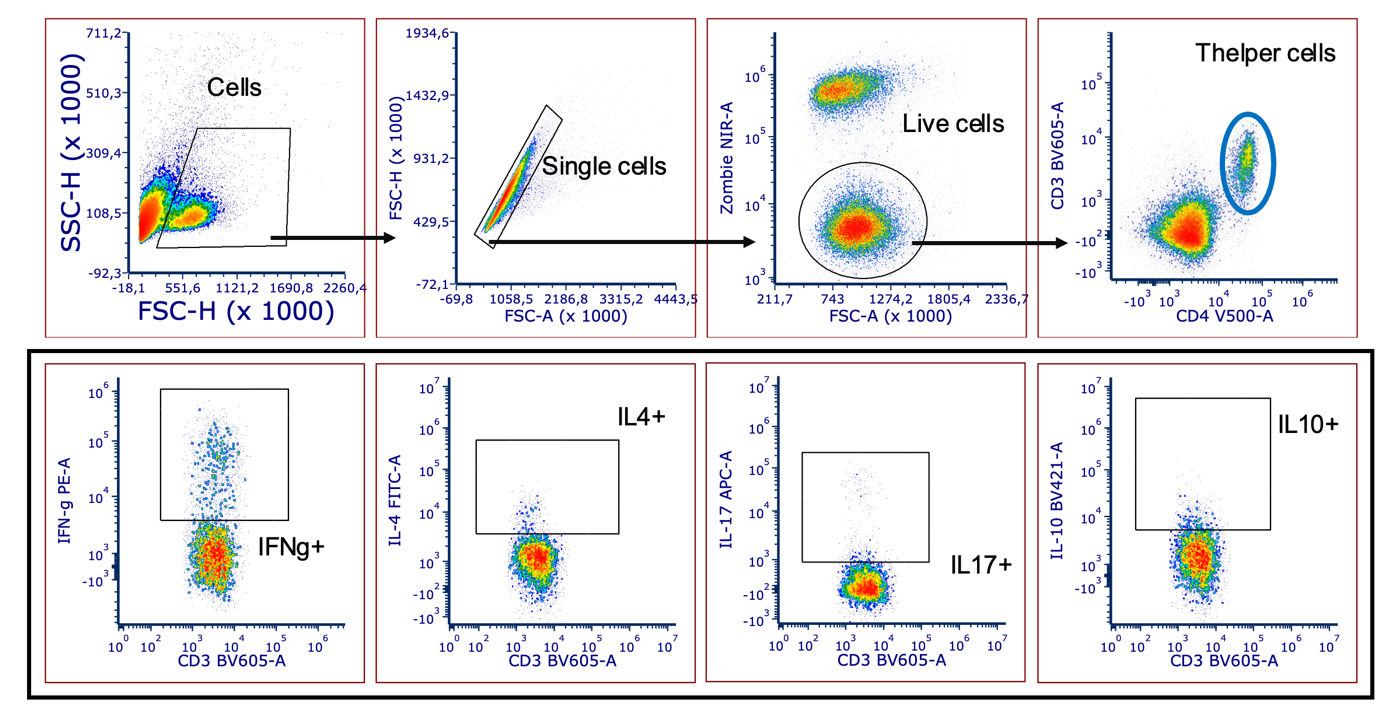


**Supplementary Figure 4.** Gating strategy for the determination of cytokine production by splenic T helper cell upon stimulation. Cells were selected based on their forward and side scatter properties. Next, single cells were selected, whereafter live cells (Zombie NIR^-^) were selected. T helper cells (CD3^+^CD4^+^) were selected within the live cell population. Cytokine production was assessed by gating IFN-ϒ^+^, IL-4^+^, IL-17A^+^ and IL-10^+^ T helper cells (these gates were set on non-stimulated matched samples).


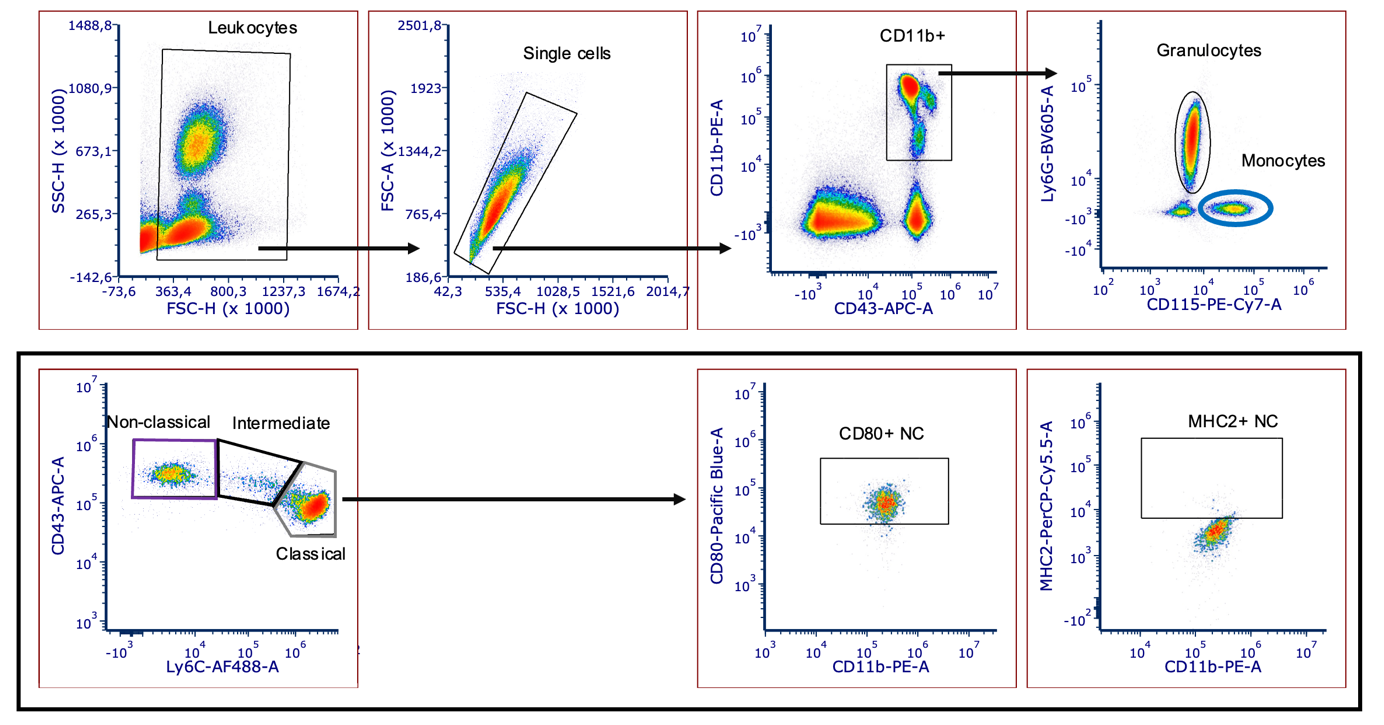


**Supplementary Figure 5.** Gating strategy for the determination of monocyte subsets and their activation status in the blood. Cells were selected based on their characteristic forward and side scatter properties. Next single cells were selected, whereafter myeloid cells (CD11b^+^) were selected. Next monocytes (Ly6G^-^CD115^+^) were selected. Subsequently, non-classical (CD43^+^Ly6C^low^), intermediate (CD43^+^Ly6C^int^) and classical (CD43^low^Ly6C^high^) monocytes were selected. Next, we determined CD80 and MHCII expression on each of these subsets (an example is shown for non-classical monocytes) using” Fluorescence Minus One” controls.


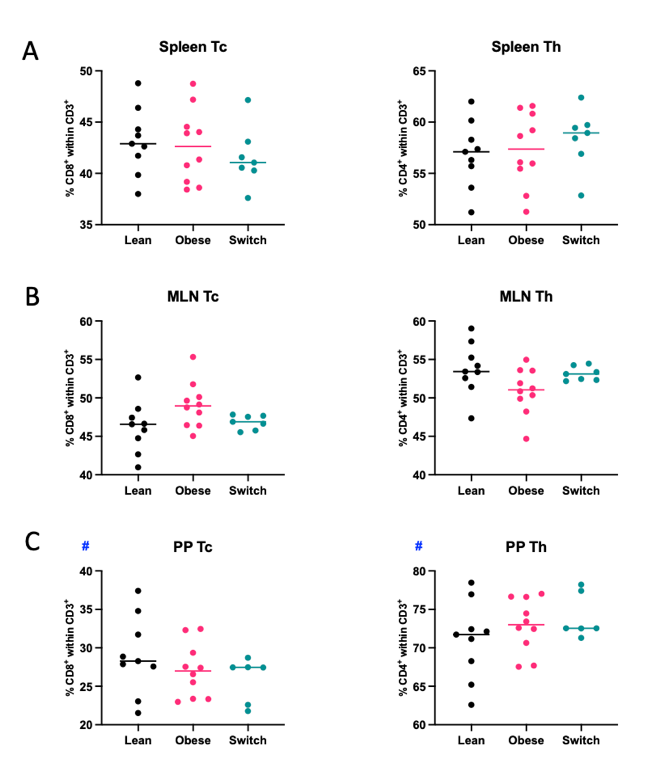


**Supplementary Figure 6.** Frequencies of cytotoxic T cells (Tc; CD3^+^CD8^+^) and T helper cells (Th; CD3^+^CD4^+^) in the **(A)** spleen, **(B)** mesenteric lymph nodes (MLN) and **(C)** Peyer’s patches (PP) of lean, obese and switch mice at day 18 of pregnancy. Data are presented as individual values and median. One-way ANOVA followed by Šídák's multiple comparisons test. # = data were log-transformed before statistical analysis. Lean mice: n = 9, obese mice: n = 10 and switch mice: n = 6/7.

**Supplementary Figure 7.** Shannon diversity index in the feces of lean and obese mice measured before and during pregnancy on day 7 (E7), day 14 (E14) and day 18 (E18). Data are presented as individual values and median. Kruskal-Wallis-test followed by Dunn’s multiple comparisons test. Lean mice before: n = 7, lean mice E7: n = 8, lean mice E14: n = 9, lean mice E18: n = 9, obese mice before: n =6, obese mice E7: n = 7, obese mice E14: n = n = 7 and obese mice E18: n = 8.

**Supplementary Table 4:** PERMANOVA results for obese and diet switch mice before the diet switch (E7 of pregnancy) and after the diet switch (E14 and E18 of pregnancy)

| **Obese versus switch mice** | **P-value** | **Significance** |
| --- | --- | --- |
| E7 of pregnancy: before the diet switch | > 0.05 | ns |
| E14 of pregnancy: after the diet switch | < 0.05 | * |
| E18 of pregnancy: after the diet switch | < 0.01 | ** |

**Supplementary Table 5:** Bacterial phyla and genera that significantly differed in the gut of obese and lean mice, before and during pregnancy on days 7, 14 and 18. Kruskal-Wallis-test followed by Dunn’s multiple comparisons test.

|  | Comparisons (p-value) | | | | Relative mean abundance | | | | | | | |
| --- | --- | --- | --- | --- | --- | --- | --- | --- | --- | --- | --- | --- |
|  | **Lean vs. Obese before** | **Lean vs. Obese E7** | **Lean vs. Obese E14** | **Lean vs. Obese E18** | **Lean before** | **Lean E7** | **Lean E14** | **Lean E18** | **Obese before** | **Obese E7** | **Obese E14** | **Obese E18** |
| **Phylum** |  |  |  |  |  |  |  |  |  |  |  |  |
| Actinobacteria | 0,1323 | **0,0034** | 0,2484 | **0,0079** | 3,75E-02 | 6,49E-02 | 5,36E-02 | 9,71E-02 | 6,57E-03 | 7,32E-03 | 1,70E-02 | 1,57E-02 |
| Verrucomicrobia | **0,0011** | **0,0004** | 0,1521 | 0,7725 | 3,36E-03 | 5,05E-03 | 6,24E-04 | 1,63E-04 | 5,54E-06 | 0,00E+00 | 3,32E-05 | 1,25E-05 |
| **Genus** |  |  |  |  |  |  |  |  |  |  |  |  |
| Bifidobacterium | 0,0781 | **0,0010** | **0,0077** | **0,0003** | 3,35E-02 | 6,20E-02 | 4,98E-02 | 9,36E-02 | 5,49E-04 | 5,08E-04 | 1,09E-03 | 8,19E-04 |
| Leifsonia | **0,0224** | >0,9999 | >0,9999 | >0,9999 | 0,00E+00 | 4,16E-06 | 0,00E+00 | 0,00E+00 | 2,22E-05 | 0,00E+00 | 0,00E+00 | 0,00E+00 |
| Olsenella | **0,0014** | **0,0307** | >0,9999 | 0,5703 | 2,63E-03 | 1,43E-03 | 1,65E-03 | 1,39E-03 | 1,66E-05 | 9,50E-06 | 9,45E-04 | 4,82E-04 |
| Adlercreutzia | 0,2000 | 0,0643 | **0,0026** | **0,0059** | 1,35E-03 | 1,41E-03 | 1,97E-03 | 2,00E-03 | 5,34E-03 | 6,44E-03 | 1,46E-02 | 1,38E-02 |
| Duncaniella | 0,1753 | **0,0034** | >0,9999 | >0,9999 | 1,01E-03 | 3,83E-03 | 5,43E-04 | 3,99E-04 | 3,49E-04 | 4,61E-04 | 3,18E-04 | 6,90E-04 |
| Muribaculum | **0,0137** | **0,0005** | 0,8663 | 0,0552 | 1,19E-04 | 1,99E-04 | 2,96E-05 | 5,54E-05 | 0,00E+00 | 0,00E+00 | 0,00E+00 | 4,16E-06 |
| Paramuribaculum | 0,0879 | **0,0186** | **0,0409** | **0,0242** | 4,43E-03 | 4,53E-03 | 2,63E-03 | 4,27E-03 | 8,37E-04 | 5,13E-04 | 2,09E-04 | 5,28E-04 |
| Parabacteroides | **0,0064** | **0,0024** | 0,1228 | 0,0854 | 2,59E-03 | 3,95E-03 | 2,00E-03 | 2,65E-03 | 2,71E-04 | 3,28E-04 | 1,19E-04 | 4,45E-04 |
| Ligilactobacillus | >0,9999 | >0,9999 | >0,9999 | **0,0236** | 1,43E-01 | 1,95E-01 | 1,58E-01 | 8,89E-02 | 1,90E-01 | 2,29E-01 | 1,48E-01 | 2,54E-01 |
| Lactococcus | **0,0208** | 0,0602 | **0,0006** | **0,0160** | 6,16E-02 | 8,02E-02 | 5,07E-02 | 6,56E-02 | 1,92E-01 | 1,88E-01 | 2,37E-01 | 2,09E-01 |
| Christensenella | 0,3747 | **0,0076** | **0,0007** | **0,0023** | 6,08E-04 | 3,86E-04 | 7,42E-04 | 6,76E-04 | 2,22E-04 | 4,27E-05 | 6,65E-05 | 9,56E-05 |
| Eubacterium | **0,0049** | **0,0448** | 0,7358 | >0,9999 | 3,06E-03 | 2,06E-03 | 2,44E-04 | 3,69E-06 | 1,22E-04 | 5,70E-04 | 1,23E-04 | 1,36E-03 |
| Acetatifactor | **0,0063** | **0,0350** | >0,9999 | 0,1393 | 4,51E-04 | 7,65E-04 | 9,05E-04 | 1,07E-03 | 1,25E-02 | 6,47E-03 | 1,15E-03 | 3,09E-03 |
| Coprococcus | 0,0701 | **0,0356** | >0,9999 | 0,6347 | 3,80E-05 | 1,25E-05 | 1,55E-04 | 1,37E-04 | 2,05E-04 | 1,14E-04 | 1,47E-04 | 3,24E-04 |
| Lacrimispora | **0,0049** | 0,5777 | 0,1064 | >0,9999 | 0,00E+00 | 0,00E+00 | 0,00E+00 | 0,00E+00 | 6,09E-05 | 2,37E-05 | 3,32E-05 | 1,25E-05 |
| Clostridium IV | **0,0179** | **0,0006** | **0,0220** | >0,9999 | 2,04E-04 | 2,74E-04 | 9,97E-05 | 3,69E-06 | 5,54E-06 | 0,00E+00 | 0,00E+00 | 0,00E+00 |
| Amedibacillus | **0,0307** | **0,0207** | 0,9784 | 0,3629 | 1,90E-05 | 4,16E-06 | 2,22E-05 | 5,54E-05 | 3,44E-04 | 3,51E-04 | 1,38E-04 | 7,23E-04 |
| Clostridium XVIII | **0,0037** | **0,0149** | **0,0009** | **0,0040** | 4,75E-06 | 0,00E+00 | 0,00E+00 | 0,00E+00 | 1,21E-03 | 1,46E-03 | 8,98E-04 | 9,52E-04 |
| Faecalibaculum | **0,0002** | 0,0546 | 0,2099 | **0,0039** | 4,24E-01 | 3,01E-01 | 4,01E-01 | 4,63E-01 | 3,06E-03 | 3,81E-02 | 2,23E-01 | 1,36E-01 |
| Pseudescherichia | **0,0352** | >0,9999 | >0,9999 | >0,9999 | 9,50E-06 | 7,90E-05 | 1,48E-05 | 4,80E-05 | 9,97E-05 | 5,70E-05 | 3,32E-05 | 2,08E-05 |
| Pseudomonas | 0,2285 | >0,9999 | 0,8318 | **0,0199** | 9,50E-06 | 2,49E-05 | 1,48E-05 | 4,06E-05 | 9,42E-05 | 1,90E-05 | 0,00E+00 | 0,00E+00 |
| Stenotrophomonas | **0,0429** | >0,9999 | >0,9999 | 0,1247 | 9,50E-06 | 4,16E-06 | 0,00E+00 | 1,48E-05 | 4,99E-05 | 9,50E-06 | 0,00E+00 | 0,00E+00 |
| Akkermansia | **0,0011** | **0,0004** | 0,1521 | 0,7725 | 3,36E-03 | 5,05E-03 | 6,24E-04 | 1,63E-04 | 5,54E-06 | 0,00E+00 | 3,32E-05 | 1,25E-05 |

**Supplementary Table 6**: Bacterial phyla and genera that significantly differed in the gut of switch and obese mice, during pregnancy on days 14 and 18. Kruskal-Wallis-test followed by Dunn’s multiple comparisons test.

|  | Comparisons (p-value) | | Relative mean abundance | | | |
| --- | --- | --- | --- | --- | --- | --- |
|  | **Switch vs.**  **obese E14** | **Switch vs. obese E18** | **Obese E14** | **Obese E18** | **Switch E14** | **Switch E18** |
| **Phylum** |  |  |  |  |  |  |
| Firmicutes | **0,0383** | 0,8930 | 9,26E-01 | 8,57E-01 | 8,11E-01 | 7,86E-01 |
| **Genus** |  |  |  |  |  |  |
| Corynebacterium | **0,0274** | >0,9999 | 3,32E-05 | 4,16E-06 | 0,00E+00 | 0,00E+00 |
| Adlercreutzia | **0,0004** | **0,0019** | 1,46E-02 | 1,38E-02 | 3,01E-03 | 3,10E-03 |
| Bacteroides | **0,0023** | 0,1066 | 6,79E-04 | 6,96E-03 | 2,48E-02 | 2,21E-02 |
| Duncaniella | **0,0317** | >0,9999 | 3,18E-04 | 6,90E-04 | 1,64E-03 | 6,29E-04 |
| Muribaculum | **0,0001** | **0,0013** | 0,00E+00 | 4,16E-06 | 9,24E-04 | 4,14E-04 |
| Staphylococcus | **0,0278** | **0,014** | 0,00E+00 | 1,66E-05 | 6,80E-04 | 5,75E-03 |
| Enterococcus | **0,0095** | 0,3638 | 1,20E-02 | 4,91E-03 | 1,67E-04 | 4,31E-04 |
| Ligilactobacillus | **0,022** | **0,0231** | 1,48E-01 | 2,54E-01 | 1,91E-02 | 6,88E-02 |
| Lactococcus | **0,0005** | **0,0006** | 2,37E-01 | 2,09E-01 | 2,23E-02 | 1,96E-02 |
| Streptococcus | **0,0482** | 0,0961 | 1,70E-03 | 1,55E-03 | 2,27E-04 | 2,68E-04 |
| Acetatifactor | 0,2657 | **0,0496** | 1,15E-03 | 3,09E-03 | 5,60E-03 | 1,30E-04 |
| Anaerotignum | 0,2162 | **0,0402** | 1,24E-03 | 3,09E-03 | 4,12E-03 | 3,39E-04 |
| Roseburia | >0,9999 | **0,0243** | 9,97E-05 | 6,15E-04 | 1,87E-04 | 0,00E+00 |
| Schaedlerella | **0,0309** | **0,0002** | 1,80E-03 | 3,56E-03 | 3,45E-04 | 8,78E-05 |
| Acutalibacter | **0,0278** | >0,9999 | 9,97E-05 | 3,62E-04 | 2,26E-03 | 2,50E-04 |
| Lawsonibacter | **0,0077** | >0,9999 | 1,45E-04 | 9,64E-04 | 1,09E-02 | 2,57E-04 |
| Neglecta | 0,8061 | **0,0245** | 2,53E-04 | 6,65E-04 | 9,51E-04 | 4,98E-05 |
| Faecalibaculum | 0,5671 | **0,0264** | 1,95E-01 | 1,27E-01 | 3,61E-01 | 5,74E-01 |
